# Synthesis and dynamics studies of barbituric acid derivatives as urease inhibitors

**DOI:** 10.1186/s13065-015-0140-1

**Published:** 2015-11-17

**Authors:** Assem Barakat, Abdullah Mohammed Al-Majid, Gehad Lotfy, Fiza Arshad, Sammer Yousuf, M. Iqbal Choudhary, Sajda Ashraf, Zaheer Ul-Haq

**Affiliations:** Department of Chemistry, College of Science, King Saud University, P.O. Box 2455, Riyadh, 11451 Saudi Arabia; Department of Chemistry, Faculty of Science, Alexandria University, P.O. Box 426, Ibrahimia, Alexandria, 21321 Egypt; Pharmaceutical Organic Chemistry Department, Faculty of Pharmacy, Suez Canal University, Ismailia, Egypt; H.E.J. Research Institute of Chemistry, International Center for Chemical and Biological Sciences, University of Karachi, Karachi, 75270 Pakistan; Dr. Panjwani Center for Molecular Medicine and Drug Research, International Center for Chemical and Biological Sciences, University of Karachi, Karachi, 75270 Pakistan

**Keywords:** Barbituric acid, Zwitterions, Urease enzyme, Urolitheasis, MD simulation and molecular docking

## Abstract

**Background:**

Discovery of potent inhibitors of urease (jack bean) enzyme is the first step in the development of drugs against diseases caused by ureolytic enzyme.

**Results:**

Thirty-two derivatives of barbituric acid as zwitterionic adducts of diethyl ammonium salts were synthesized. All synthesized compounds (**4a**–**z** and **5a**–**s)** were screened for their in vitro inhibition potential against urease enzyme (jack bean urease). The compounds **4i** (IC_50_ = 17.6 ± 0.23 µM) and **5l** (IC_50_ = 17.2 ± 0.44 µM) were found to be the most active members of the series, and showed several fold more urease inhibition activity than the standard compound thiourea (IC_50_ = 21.2 ± 1.3 µM). Whereas, compounds **4a**–**b**, **4d**–**e**, **4g**–**h**, **4j**–**4r**, **4x**, **4z**, **5b**, **5e**, **5k**, **5n**–**5q** having IC_50_ values in the range of 22.7 ± 0.20 µM–43.8 ± 0.33 µM, were also found as potent urease inhibitors. Furthermore, Molecular Dynamics simulation and molecular docking studies were carried out to analyze the binding mode of barbituric acid derivatives using MOE. During MD simulation enol form is found to be more stable over its keto form due to their coordination with catalytic Nickel ion of Urease. Additionally, structural–activity relationship using automated docking method was applied where the compounds with high biological activity are deeply buried within the binding pocket of urease. As multiple hydrophilic crucial interactions with Ala169, KCX219, Asp362 and Ala366 stabilize the compound within the binding site, thus contributing greater activity.

**Conclusions:**

This research study is useful for the discovery of economically, efficient viable new drug against infectious diseases.

**Graphical abstract:**
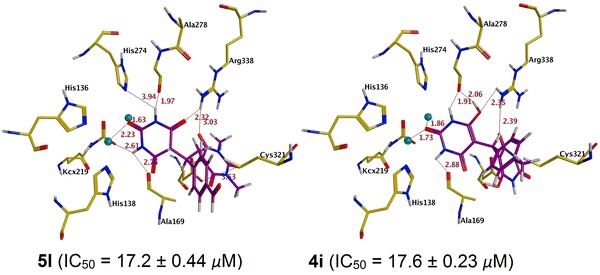
STD. Thiourea (IC_50_ = 21.2 ± 1.3 µM)

**Electronic supplementary material:**

The online version of this article (doi:10.1186/s13065-015-0140-1) contains supplementary material, which is available to authorized users.

## Background

Urease is a nickel containing enzyme produced by plants, fungi, algae, and bacteria. It is involved in nitrogen turnover and in crop fertilization, as well as in human and animal pathologies. Urease catalyse the hydrolysis of urea in its ammonia and carbon dioxide. Beside its medical, ecological and economical significances as urease has historical significances as it was the first enzyme to be crystallised in 1926 by Sumner [1–3]. Since its discovery in plants [4], *Canavalia ensiformis* (*Fabaceae*) urease has been exhaustively investigated [5]. Its activity is strictly dependent on nickel ions (Ni^2+^) [6]. The first X-ray diffraction based structure of a urease was reported by Jabri and coworkers in 1995 from *Klebsiella aerogenes* [7]. Later on, other structures for ureases from *Bacillus pasteurii* [8], *Helicobacter pylori* [9] and *C. ensiformis* [10] were reported. The elucidation of the urease structure from a legume (jack bean) was crucial to better understand the requirements for ureolytic activity of this class of enzymes in different organisms [10] were reported.

Urease enzyme is a virulence factor in certain human and animal ailments. It contributes to the development of kidney stones, pyelonephritis, peptic ulcers leading to gastric cancers, and other diseases [11]. It also causes the pathogenesis of hepatic coma urolithiasis, hepatic encephalopathy, pyelonephritis, ammonia and urinary catheter encrustation [12, 13]. The gastric cancer [14, 15] is the fourth most common cancer and the second most common cause of cancer-related deaths worldwide [16]. It is often resulted from pathologies due to *Helicobacter pylori*. Urease lets bacteria to persist at the low pH of the stomach during colonization and lead to pathogenesis of gastric and peptic ulcers which in the long run may cause cancer [17]. The treatment of infection caused by ureolytic bacteria with antimicrobials, however, often proved to be unsuccessful [13]. The barbiturates possessed a wide range of pharmacological applications, such as anticonvulsant, sedative, anxiolytic, urease inhibition [18], antifungal [19], antimicrobial [20, 21], antitumor, antiviral [13, 22] anti tuberculosis [23], mushroom tyrosinase inhibition [24], radio-sensitization [25], anti-inflammatory, anticancer [26], anesthetic [27], diaminopimelate aminotransferase inhibition [28], and anti-proliferative activities [29].

Based on the therapeutic and pharmacological significances of urease inhibition, our research group is involved in the search of simple but biologically interesting molecules that are easy to synthesize in just fewer steps with high yields. This type of chemistry is easily adopted by the pharmaceutical industry for commercialization. Previously, our research group reported zwitterionic adduct derived from barbituric acid as NO scavenger [30]. In view of these studies; the combined use of green synthetic technology for the high yield production of novel pharmacophoric barbituric acid derivatives and their systematic evalution of biological activities as urease inhibition is discussed in this paper.

## Methods

### General

All chemicals were purchased from Sigma-Aldrich, Fluka etc., and were used without further purification, unless otherwise stated. All melting points were measured on a Gallenkamp melting point apparatus in open glass capillaries and are uncorrected. IR Spectra were measured as KBr pellets on a Nicolet 6700 FT-IR spectrophotometer. The NMR spectra were recorded on a Varian Mercury Jeol-400 NMR spectrometer. ^1^H-NMR (400 MHz), and ^13^C-NMR (100 MHz) were run in deuterated chloroform (CDCl_3_). Chemical shifts (*δ*) are referred in terms of *ppm* and *J*-coupling constants are given in *Hz*. Mass spectra were recorded on a Jeol JMS-600 H. Elemental analysis was carried out on Elmer 2400 Elemental Analyzer in CHN mode

### Synthesis of 4 and 5 (GP1)

A mixture of **1** (3 mmol) and aldehyde **2** (1.5 mmol), as well as Et_2_NH (1.5 mmol, 155 μL) were placed in 3 mL of degassed H_2_O. The reaction mixture was kept at rt up to 5 h under stirring. After completion of the reaction, monitored by TLC, the solid product was filtered, washed with ether (3 × 20 mL) and dried to obtain pure products **4** and **5.**

#### 4-(bis(6-Hydroxy-1,3-dimethyl-2,4-dioxo-1,2,3,4-tetrahydropyrimidin-5-yl)methyl)benzaldehyde diethylaminium salt **4a**

**4a**, as colorless crystal (1.5 g, 2.76 mmol, 92 %). IR (cm^−1^): 3450, 3000, 2872, 1670, 1582, 1510, 1466, 1384, 1339; ^1^H-NMR (CDCl_3_, 400 MHz) 17.58 (s, 1H, OH), 9.90(s, 1H, CHO), 7.73 (d, 2H, *J* = 8.0 Hz, Ph), 7.29 (d, 2H, *J* = 8.0 Hz, Ph), 5.93(s, 1H, benzyl-H), 3.33 (s, 12H, 4CH_3_), 3.06 (q, 4H, *J* = 7.3 Hz, C*H*_2_CH_3_), 1.27 (t, 6H, *J* = 7.3 Hz, CH_2_C*H*_3_); ^13^C-NMR (100 MHz, CDCl_3_): *δ* = 192.2, 165.3, 164.4, 151.7, 150.3, 134.3, 129.9, 127.3, 91.7, 42.2, 35.1, 29.0, 28.7, 11.5; Anal. for C_24_H_31_N_5_O_7_; Calcd: C, 57.48; H, 6.23; N, 13.96; Found:C, 57.50; H, 6.25; N, 14.00; LC/MS (ESI): *m*/*z* = 501.53 [M]^+^.

#### 5,5′-(3-Tolylmethylene)bis(1,3-dimethylpyrimidine-2,4,6(1H,3H,5H)-trione) diethylaminium salt **4b**

**4b**; rose-colored crystalline materials. m.p.: 135 °C; (97 %, 1.41 g, 2.91 mmol). IR (KBr, cm^−1^): 3455, 3201, 2988, 1693, 1667, 1611, 1573, 1443; ^1^H-NMR (400 MHz, CDCl_3_): *δ*17.62 (s, 1H, OH), 7.10 (t, 1H, *J* = 7.3 Hz, Ph), 6.92 (d, 1H, *J* = 7.3 Hz, Ph), 6.88 (d, 1H, *J* = 7.3 Hz, Ph), 5.82(s, 1H, benzyl-H), 3.32 (s, 12H, 4CH_3_), 3.01 (q, 4H, *J* = 7.3 Hz, C*H*_2_CH_3_), 2.25 (s, 3H, CH_3_), 1.26 (t, 6H, *J* = 7.3 Hz, CH_2_C*H*_3_); ^13^C-NMR (100 MHz, CDCl_3_): *δ* = 165.3, 164.4, 151.8, 141.7, 137.4, 127.9, 127.1, 126.4, 123.6, 92.1, 42.0, 34.4, 28.9, 28.6, 21.8, 11.4; Anal. for C_24_H_35_N_5_O_6_; Calcd: C, 59.12; H, 6.82; N, 14.36; Found: C, 59.13; H, 6.81; N, 14.35; LC/MS (ESI): *m*/*z* = 487[M]^+^.

#### 5,5′-((4-Nitrophenyl)methylene)bis(1,3-dimethylpyrimidine-2,4,6(1H,3H,5H)-trione)diethylaminium salt **4c**

**4c**; a yellow powder; m.p.: 195 °C; (87 %, 1.35 g, 2.61 mmol); IR (KBr, cm^−1^): 3453, 3205, 2987, 2904, 1675, 1608, 1576, 1511, 1438, 1343, 1254;^1^H-NMR (400 MHz, CDCl_3_): *δ*17.58 (s, 1H, OH), 8.08 (d, 2H, *J* = 8.8 Hz, Ph), 7.29 (d, 2H, *J* = 8.8 Hz, Ph), 5.95(s, 1H, benzyl-H), 3.34 (s, 12H, 4CH_3_), 3.07 (q, 4H, *J* = 7.3 Hz, C*H*_2_CH_3_), 1.29 (t, 6H, *J* = 7.3 Hz, CH_2_C*H*_3_); ^13^C-NMR (100 MHz, CDCl_3_): *δ* = 165.2, 164.4, 151.6, 150.8, 146.1, 127.5, 123.5, 91.4, 42.2, 34.9, 28.9, 28.7, 11.5; Anal. for C_23_H_30_N_6_O_8_; Calcd: C, 53.28; H, 5.83; N, 16.21; Found: C, 53.29; H, 5.85; N, 16.23; LC/MS (ESI): *m*/*z* = 518[M]^+^.

A suitable crystal for X-ray diffraction analysis was obtained from DCM/Et_2_O after 24 h. CCDC-1001798 contains the supplementary crystallographic data for this compound (Additional file 1).

#### 5,5′-((4-Methoxyphenyl)methylene)bis(1,3-dimethylpyrimidine-2,4,6(1H,3H,5H)-trione) diethylaminium salt **4d**

**4d**; rose-colored crystalline materials; m.p.: 160 °C; (90 %, 1.35 g, 2.7 mmol). IR (KBr, cm^−1^): 3445, 3195, 2977, 2836, 1689, 1664, 1613, 1504, 1447, 1378, 1242; ^1^H-NMR (400 MHz, CDCl_3_): *δ*17.67 (s, 1H, OH), 7.01 (d, 2H, *J* = 8.8 Hz, Ph), 6.75 (d, 2H, *J* = 8.8 Hz, Ph), 5.79(s, 1H, benzyl-H), 3.33 (s, 12H, 4CH_3_), 2.99 (q, 4H, *J* = 7.3 Hz, C*H*_2_CH_3_), 1.26 (t, 6H, *J* = 7.3 Hz, CH_2_C*H*_3_); ^13^C-NMR (100 MHz, CDCl_3_): *δ* = 165.3, 164.3, 157.4, 151.7, 133.6, 132.0, 127.4, 114.3, 92.1, 55.6, 42.1, 33.8, 28.9, 11.5; Anal. for C_24_H_33_N_5_O_7_; Calcd: C, 57.25; H, 6.61; N, 13.91; Found: C, 57.26; H, 6.61; N, 13.90; LC/MS (ESI): *m*/*z* = 503[M]^+^.

#### 5,5′-((3-Bromophenyl)methylene)bis(1,3-dimethylpyrimidine-2,4,6(1H,3H,5H)-trione) diethylaminium salt **4e**

**4e**; colorless crystalline materials; m.p.: 169 °C; (92 %, 1.5 g, 2.76 mmol). IR (KBr, cm^−1^): 3450, 3120, 2982, 1694, 1667, 1615, 1577, 1445, 1250; ^1^H-NMR (400 MHz, CDCl_3_): *δ*17.63 (s, 1H, OH), 7.22 (d, 1H, *J* = 7.3 Hz, Ph), 7.19 (s, 1H,Ph), 7.07 (d, 1H, *J* = 7.3 Hz, Ph), 7.05 (d, 1H, *J* = 7.3 Hz, Ph), 5.84(s, 1H, benzyl-H), 3.34 (s, 6H, 2CH_3_), 3.32 (s, 6H, 2CH_3_), 3.02 (q, 4H, *J* = 7.3 Hz, C*H*_2_CH_3_), 1.27 (t, 6H, *J* = 7.3 Hz, CH_2_C*H*_3_); ^13^C-NMR (100 MHz, CDCl_3_): *δ* = 165.2, 164.4, 151.7, 144.7, 129.7,129.6, 128.7, 125.3, 91.5, 42.1, 34.4, 28.9, 28.7, 11.5; Anal. for C_23_H_30_BrN_5_O_6_; Calcd: C, 50.01; H, 5.47; Br, 14.46; N, 12.68; Found: C, 50.03; H, 5.48; Br, 14.47; N, 12.71; LC/MS (ESI): *m*/*z* = 552[M]^+^.

A suitable crystal for X-ray diffraction analysis was obtained from DCM/Et_2_O after 24 h. CCDC-1001799 contains the supplementary crystallographic data for this compound.

#### 5,5′-((4-hydroxyphenyl)methylene)bis(6-hydroxy-1,3-dimethylpyrimidine-2,4(1H,3H)-dione) diethylaminium salt **4f**

**4f**; a yellow powder; m.p.: 180 °C; (88 %, 1.3 g, 2.64 mmol); IR (KBr, cm^−1^): 3458, 3200, 2980, 2904, 1677, 1620, 1572, 1511, 1438, 1343, 1254;^1^H-NMR (400 MHz, CDCl_3_): *δ*17.62 (s, 1H, OH), 7.31 (d, 2H, *J* = 8.8 Hz, Ph), 6.99 (d, 2H, *J* = 8.8 Hz, Ph), 5.79(s, 1H, benzyl-H), 3.33 (s, 12H, 4CH_3_), 3.03 (q, 4H, *J* = 7.3 Hz, C*H*_2_CH_3_), 1.27 (t, 6H, *J* = 7.3 Hz, CH_2_C*H*_3_); ^13^C-NMR (100 MHz, CDCl_3_): *δ* = 165.3, 164.4, 151.7, 141.1, 131.2, 128.5, 119.3, 91.7, 42.1, 34.2, 28.9, 28.7, 11.5; Anal. for C_23_H_31_N_5_O_7_; Calcd: C, 56.43; H, 6.38; N, 14.31; Found: C, 56.44; H, 6.36; N, 14.30; LC/MS (ESI): *m*/*z* = 489.52 [M]^+^.

#### 5,5′-(p-Tolylmethylene)bis(1,3-dimethylpyrimidine-2,4,6(1H,3H,5H)-trione) diethylaminium salt **4g**

**4g**; colorless needle materials; m.p.: 152 °C; (97 %, 1.41 g, 2.91 mmol). IR (KBr, cm^−1^): 3455, 3210, 2984, 2820, 1560, 1449, 1359; ^1^H-NMR (400 MHz, CDCl_3_): *δ*17.64 (s, 1H, OH), 6.99–6.96 (m, 4H, Ph), 5.80(s, 1H, benzyl-H), 3.32 (s, 12H, 4CH_3_), 3.03 (q, 4H, *J* = 7.3 Hz, C*H*_2_CH_3_), 2.25 (s, 3H, CH_3_), 1.28 (t, 6H, *J* = 7.3 Hz, CH_2_C*H*_3_); ^13^C-NMR (100 MHz, CDCl_3_): *δ* = 165.3, 164.3, 151.8, 138.6, 134.8, 128.9, 126.3, 92.1, 42.0, 34.2, 28.9, 28.6, 21.0, 11.4; Anal. for C_24_H_35_N_5_O_6_; Calcd: C, 59.12; H, 6.82; N, 14.36; Found: C,59.13; H, 6.81; N, 14.35; LC/MS (ESI): *m*/*z* = 487[M]^+^.

A suitable crystal for X-ray diffraction analysis was obtained from DCM/Et_2_O after 24 h. CCDC-957025 contains the supplementary crystallographic data for this compound.

#### 5,5′-(Naphthalen-2-ylmethylene)bis(1,3-dimethylpyrimidine-2,4,6(1H,3H,5H)-trione) diethylaminium salt **4h**

**4h**; beige powder; m.p.: 146 °C; (94 %, 1.47 g, 2.82 mmol). IR (KBr, cm^−1^): 3454, 3200, 2967, 1668, 1585, 1438, 1250;^1^H-NMR (400 MHz, CDCl_3_): *δ*17.33 (s, 1H, OH), 8.10 (d, 2H, *J* = 8.8 Hz, naphthyl-H), 7.99 (d, 2H, *J* = 8.8 Hz, naphthyl-H), 7.92 (d, 2H, *J* = 8.8 Hz, naphthyl-H), 7.90 (d, 2H, *J* = 8.8 Hz, naphthyl-H), 7.84 (d, 2H, *J* = 8.8 Hz, naphthyl-H), 7.68–7.38 (m, 3H,naphthyl-H), 6.37(s, 1H, benzyl-H), 3.39 (s, 12H, 4CH_3_), 3.01 (q, 4H, *J* = 7.3 Hz, C*H*_2_CH_3_), 1.30 (t, 6H, *J* = 7.3 Hz, CH_2_C*H*_3_); ^13^C-NMR (100 MHz, CDCl_3_): *δ* = 164.9, 151.7, 136.8, 135.3, 134.3, 131.5, 129.1, 128.5, 127.0, 125.2 124.9, 123.8, 93.2, 41.8, 33.2, 28.8, 11.4; Anal. for C_27_H_33_N_5_O_6_; Calcd: C, 61.94; H, 6.35; N, 13.38; Found: C, 61.95; H, 6.34; N, 13.40; LC/MS (ESI): *m*/*z* = 523 [M]^+^.

#### 5,5′-(p-Tolylmethylene)bis(6-hydroxypyrimidine-2,4(1H,3H)-dione) diethylaminium salt **4i**

**4i**; white powder; m.p.: 205 C; (95 %; 1.22 g, 2.85 mmol); IR (KBr, cm^−1^): 3459, 3120, 2978, 2811, 1689, 1612, 1325, 1252; ^1^H-NMR (400 MHz, DMSO-*d*_*6*_): *δ*17.18 (s, 1H, OH), 10.09 (bs, 4H, NH), 6.93 (m, 4H, Ph), 5.90(s, 1H, benzyl-H), 2.79 (q, 4H, *J* = 7.3 Hz, C*H*_2_CH_3_), 2.20 (s, 3H, CH_3_), 1.07 (t, 6H, *J* = 7.3 Hz, CH_2_C*H*_3_); ^13^C-NMR (100 MHz, DMSO-*d*_*6*_): *δ* = 164.8, 164.1, 151.3, 142.1, 133.5, 128.5, 127.1, 91.6, 42.6, 30.6, 21.1, 13.0; Anal. for C_20_H_25_N_5_O_6_; Calcd: C, 55.68; H, 5.84; N, 16.23; Found: C, 55.67; H, 5.83; N, 16.22; LC/MS (ESI): *m/z* = 431[M]^+^.

#### 5,5′-((4-Chlorophenyl)methylene)bis(6-hydroxypyrimidine-2,4(1H,3H)-dione) diethylaminium salt **4j**

**4j**; a white powder; m.p.: 221 °C; (95 %, 1.28 g, 2.85 mmol); IR (KBr, cm^−1^): 3435, 3185, 2978, 2830, 1677, 1548, 1448, 1345, 1250;^1^H-NMR (400 MHz, DMSO-*d*_*6*_): *δ*17.17 (s, 1H, OH), 10.00 (bs, 4H, NH), 7.18 (m, 4H, Ph), 5.93(s, 1H, benzyl-H), 2.88 (q, 4H, *J* = 7.3 Hz, C*H*_2_CH_3_), 1.12 (t, 6H, *J* = 7.3 Hz, CH_2_C*H*_3_); ^13^C-NMR (100 MHz, DMSO-*d*_*6*_): *δ* = 164.7, 164.0, 151.2, 144.6, 133.5, 129.9, 129.1, 127.8, 91.3, 42.1, 30.7, 11.8; Anal. for C_19_H_22_ClN_5_O_6_; Calcd C, 50.50; H, 4.91; Cl, 7.85; N, 15.50; Found: C, 50.51; H, 4.90; Cl, 7.83; N, 15.51; LC/MS (ESI): *m*/*z* = 451[M]^+^.

#### 5,5′-((4-Methoxyphenyl)methylene)bis(6-hydroxypyrimidine-2,4(1H,3H)-dione) diethylaminium salt **4K**

**4k**; a beige powder; m.p.: 195 °C; (91 %, 1.22 g, 2.73 mmol); IR (KBr, cm^−1^): 3449, 3190, 2991, 2835, 1688, 1592, 1505, 1383, 1247;^1^H-NMR (400 MHz, DMSO-*d*_*6*_): *δ*17.26 (s, 1H, OH), 9.99 (bs, 4H, NH), 6.92 (d, 2H, *J* = 8.0 Hz, Ph), 6.72 (d, 2H, *J* = 8.0 Hz, Ph), 5.88(s, 1H, benzyl-H), 2.90 (q, 4H, *J* = 7.3 Hz, C*H*_2_CH_3_), 1.14 (t, 6H, *J* = 7.3 Hz, CH_2_C*H*_3_); ^13^C-NMR (100 MHz, DMSO-*d*_*6*_): *δ* = 164.6, 164.0, 157.0, 151.2, 137.2, 132.4, 115.1, 91.7, 55.4, 42.1, 30.7, 11.6; Anal. for C_20_H_25_N_5_O_7_; Calcd C, 53.69; H, 5.63; N, 15.65; Found: C, 53.69; H, 5.63; N, 15.66; LC/MS (ESI): *m*/*z* = 447[M]^+^.

#### 5,5′-(Naphthalen-2-ylmethylene)bis(6-hydroxypyrimidine-2,4(1H,3H)-dione) diethylaminium salt **4l**

**4** **l**; a beige powder, m.p.: 192 °C; (93 %, 1.3 g, 2.79 mmol); IR (KBr, cm^−1^): 3459, 3208, 2994, 1677, 1579, 1448, 1386, 1354;^1^H-NMR (400 MHz, DMSO-*d*_*6*_): *δ*16.92 (s, 1H, OH), 10.41 (bs, 4H, NH), 8.13 (d, 1H, *J* = 8.8 Hz, naphthyl), 7.81(d, 1H, *J* = 8.8 Hz, naphthyl), 7.63 (d, 1H, *J* = 8.8 Hz, naphthyl), 7.38–7.32 (m, 4H, naphthyl), 6.46(s, 1H, benzyl-H), 2.79 (q, 4H, *J* = 7.3 Hz, C*H*_2_CH_3_), 1.08 (t, 6H, *J* = 7.3 Hz, CH_2_C*H*_3_); ^13^C-NMR (100 MHz, DMSO-*d*_*6*_): *δ* = 164.9, 151.1,141.5, 135.8, 134.0,132.4, 129.3, 128.7, 126.0,125.8, 125.5, 125.2, 124.9, 123.8, 92.3, 42.5, 29.7, 12.7; Anal. for C_23_H_25_N_5_O_6_; Calcd C, 59.09; H, 5.39; N, 14.98; Found: C, 59.12; H, 5.40; N, 15.01; LC/MS (ESI): *m*/*z* = 467[M]^+^.

#### 5-((2-Hydroxy-4,4-dimethyl-6-oxocyclohex-1-en-1-yl)(phenyl)methyl)-1,3-dimethyl-2,6-dioxo-1,2,3,6-tetrahydropyrimidin-4-olate diethylaminium salt **4m**

**4m**; colorless crystalline material; m.p: 159 °C; (98 %, 671 mg, 1.47 mmol). IR (KBr, *cm*^−*1*^): 3150, 2959, 1667, 1617, 1585, 1422, 1256, 1227;^1^H NMR (400 MHz, CDCl_3_): *δ* 15.28 (s, 1H, OH), 7.17–7.04(m, 5H, Ph), 5.85 (s, 1H, benzyl-H), 3.29 (s, 12H, 4CH_3_), 2.96(q, 4H, *J* = 7.3 Hz, C*H*_2_CH_3_), 2.42 (d, 2H, *J* = 5.1 Hz, CH_2_), 2.29 (m, 2H, CH_2_), 1.24(t, 6H, *J* = 7.3 Hz, CH_2_C*H*_3_), 1.14(s, 3H, CH_3_), 1.05(s, 3H, CH_3_); ^13^C NMR (100 MHz, CDCl_3_): *δ* = 192.5, 180.8, 152.5, 142.5, 128.0, 126.7, 125.1, 116.3, 90.9, 51.4, 45.9, 42.2, 33.0, 31.5, 29.6, 28.4, 27.6, 11.4; Anal. for C_25_H_35_N_3_O_5_; calcd: C, 65.62; H, 7.71; N, 9.18;Found: C, 65.61; H, 7.73; N, 9.20; LC/MS (ESI): *m*/*z* = 457 [M]^+^.

A suitable crystal for X-ray diffraction analysis was obtained from CHCl_3_/Et_2_O after 24 h. CCDC- 933624 contains the supplementary crystallographic data for this compound.

#### 5-((2-Hydroxy-4,4-dimethyl-6-oxocyclohex-1-en-1-yl)(p-tolyl)methyl)-1,3-dimethyl-2,6-dioxo-1,2,3,6-tetrahydropyrimidin-4-olate diethylaminium salt **4n**

**4n**; oily material (97 %; 685 mg, 1.45 mmol). IR (KBr, *cm*^−*1*^): 3150, 2954, 2867, 1675, 1580, 1508, 1447, 1380, 1256, 1145;^1^H NMR (400 MHz, CDCl_3_): *δ* 15.25 (s, 1H, OH), 7.00–6.93(m, 4H, Ph), 5.84 (s, 1H, benzyl-H), 3.28 (s, 12H, 4CH_3_), 2.90(q, 4H, *J* = 7.3 Hz, C*H*_2_CH_3_), 2.30 (d, 4H, *J* = 5.1 Hz, CH_2_), 2.22 (s, 3H, CH_3_), 1.20(t, 6H, *J* = 7.3 Hz, CH_2_C*H*_3_), 1.16(s, 3H, CH_3_), 1.04(s, 3H, CH_3_); ^13^C NMR (100 MHz, CDCl_3_): *δ* = 196.5, 180.1, 152.8, 140.5, 134.2, 129.8, 128.7, 126.8, 126.7, 115.6, 91.0, 51.4, 45.9, 42.5, 32.6, 31.5, 29.6, 28.4, 27.6, 20.9, 11.9; Anal. for C_26_H_37_N_3_O_5_; calcd: C, 66.22; H, 7.91; N, 8.91;Found: C, 66.24; H, 7.92; N, 8.87; LC/MS (ESI): *m*/*z* = 471 [M]^+^.

#### 5-((2-Hydroxy-4,4-dimethyl-6-oxocyclohex-1-en-1-yl)(4-methoxyphenyl)methyl)-1,3-dimethyl-2,6-dioxo-1,2,3,6-tetrahydropyrimidin-4-olate diethylaminium salt **4o**

**4o**; an oily material (92 %; 672 mg, 1.38 mmol). IR (KBr, *cm*^−*1*^): 3047, 2953, 2866, 2499, 1679, 1577, 1510, 1427, 1373, 1255, 1214;^1^H NMR (400 MHz, CDCl_3_): *δ* 15.26 (s, 1H, OH), 6.98(d, 2H, *J* = 8.0 Hz, Ph), 6.72(d, 2H, *J* = 8.0 Hz, Ph), 5.69 (s, 1H, benzyl-H), 3.71 (s, 3H, CH_3_), 3.29 (s, 12H, 4CH_3_), 2.87(q, 4H, *J* = 7.3 Hz, C*H*_2_CH_3_), 2.31 (d, 4H, *J* = 5.1 Hz, CH_2_), 1.19(t, 6H, *J* = 7.3 Hz, CH_2_C*H*_3_), 1.12(s, 3H, CH_3_), 1.03(s, 3H, CH_3_); ^13^C NMR (100 MHz, CDCl_3_): *δ* = 195.1, 187.2, 157.1, 134.5, 133.9, 127.8, 127.6, 115.6, 113.4, 55.2, 42.6, 31.5, 31.1, 27.9, 12.2; Anal. for C_26_H_37_N_3_O_6_; calcd: C, 64.05; H, 7.65; N, 8.62;Found: C, 64.11; H, 7.64; N, 8.59; LC/MS (ESI): *m*/*z* = 487 [M]^+^.

#### 5-((4-Chlorophenyl)(2-hydroxy-4,4-dimethyl-6-oxocyclohex-1-en-1-yl)methyl)-1,3-dimethyl-2,6-dioxo-1,2,3,6-tetrahydropyrimidin-4-olate diethylaminium salt **4p**

**4p**; oily material (97 %; 715 mg, 1.45 mmol). IR (KBr, cm^−1^): 3151, 2955, 2868, 2497, 1675, 1580, 1481, 1444, 1379, 1258, 1206;^1^H NMR (400 MHz, CDCl_3_): *δ* 15.02 (s, 1H, OH), 7.12–6.95(m, 4H, Ph), 5.87 (s, 1H, benzyl-H), 3.30 (s, 12H, 4CH_3_), 2.90(q, 4H, *J* = 7.3 Hz, C*H*_2_CH_3_), 2.38 (s, 4H, CH_2_), 1.20(t, 6H, *J* = 7.3 Hz, CH_2_C*H*_3_), 1.16(s, 3H, CH_3_), 1.04(s, 3H, CH_3_); ^13^C NMR (100 MHz, CDCl_3_): *δ* = 198.1, 181.0, 152.5, 141.5, 130.6, 128.3, 128.2, 128.0, 127.9, 115.2, 90.7, 65.9, 49.8, 42.3, 32.4, 31.5, 31.2, 29.6, 28.4, 27.6, 15.3, 11.4; Anal. for C_25_H_34Cl_N_3_O_5_; calcd: C, 61.03; H, 6.97; Cl, 7.21; N, 8.54;Found: C, 61.06; H, 7.00; Cl, 7.18; N, 8.57; LC/MS (ESI): *m*/*z* = 492 [M]^+^.

#### 5-((4-Bromophenyl)(2-hydroxy-4,4-dimethyl-6-oxocyclohex-1-en-1-yl)methyl)-1,3-dimethyl-2,6-dioxo-1,2,3,6-tetrahydropyrimidin-4-olate diethylaminium salt **4q**

**4q**; an oily material (95 %, 761 mg, 1.42 mmol). IR (KBr, cm^−1^): 3155, 2955, 2867, 2500, 1674, 1579, 1430, 1376, 1204;^1^H NMR (400 MHz, CDCl_3_): *δ* 15.20 (s, 1H, OH), 7.34 (d, 2H, *J* = 8.0 Hz, Ph), 6.98 (d, 2H, *J* = 8.0 Hz, Ph), 5.79 (s, 1H, benzyl-H), 3.27 (s, 12H, 4CH_3_), 2.99(q, 4H, *J* = 7.3 Hz, C*H*_2_CH_3_), 2.40 (d, 2H, *J* = 5.1 Hz, CH_2_), 2.28(m, 2H, CH_2_), 1.29(t, 6H, *J* = 7.3 Hz, CH_2_C*H*_3_), 1.18(s, 3H, CH_3_), 1.04(s, 3H, CH_3_); ^13^C NMR (100 MHz, CDCl_3_): *δ* = 199.1, 191.2, 164.8, 152.4, 142.8, 132.5, 131.0, 129.9, 128.7, 128.6, 118.9, 115.9, 90.6, 51.2, 45.8, 42.3, 32.7, 31.5, 29.5, 28.5, 28.3, 27.6, 11.4; Anal. for C_25_H_34_BrN_3_O_5_; calcd: C, 55.97; H, 6.39; Br, 14.89; N, 7.83;Found: C, 56.00; H, 6.40; Br, 14.86; N, 7.82; LC/MS (ESI): *m*/*z* = 536 [M]^+^.

#### 5-((3-Bromophenyl)(2-hydroxy-4,4-dimethyl-6-oxocyclohex-1-en-1-yl)methyl)-1,3-dimethyl-2,6-dioxo-1,2,3,6-tetrahydropyrimidin-4-olate diethylaminium salt **4r**

**4r**; oily material (93 %, 745 mg, 1.39 mmol). IR (KBr, cm^−*1*^): 3050, 2955, 2868, 2500, 1675, 1581, 1444, 1378, 1255, 1205; ^1^H NMR (400 MHz, CDCl_3_): *δ* 15.63 (s, 1H, OH), 7.22 (d, 1H, *J* = 7.3 Hz, Ph), 7.19 (s, 1H, Ph), 7.07 (d, 1H, *J* = 7.3 Hz, Ph), 7.05 (d, 1H, *J* = 7.3 Hz, Ph), 5.84 (s, 1H, benzyl-H), 3.34(s, 6H, 2CH_3_), 3.32(s, 6H, 2CH_3_), 2.98(q, 4H, *J* = 7.3 Hz, CH_2_CH_3_), 2.31 (d, 4H, *J* = 5.1 Hz, CH_2_), 1.24(t, 6H, *J* = 7.3 Hz, CH_2_C*H*_3_), 1.12(s, 3H, CH_3_), 1.03(s, 3H, CH_3_); ^13^C NMR (100 MHz, CDCl_3_): *δ* = 190.8, 186.4, 165.2, 164.4, 151.7, 144.7, 129.7,129.6, 128.7, 125.3, 91.5, 42.1, 34.4, 28.9, 28.7, 11.5; Anal. for C_25_H_34_BrN_3_O_5_; calcd: C, 55.97; H, 6.39; Br, 14.89; N, 7.83;Found: C, 56.01; H, 6.41; Br, 14.86; N, 7.84; LC/MS (ESI): *m*/*z* = 536 [M]^+^.

#### 5-((2-Hydroxy-4,4-dimethyl-6-oxocyclohex-1-en-1-yl)(1-nitrophenyl)methyl)-1,3-dimethyl-2,6-dioxo-1,2,3,6-tetrahydropyrimidin-4-olate diethylaminium salt **4s**

**4s**; a beige material; m.p: 146 °C; (92 %, 690 mg, 1.37 mmol). IR (KBr, cm^−1^): 3054, 2953, 2865, 2500, 1673, 1580, 1510, 1427, 1373, 1255, 1214;^1^H NMR (400 MHz, CDCl_3_): *δ* 15.33 (s, 1H, OH), 7.01-7.35 (m, 3H, Ph), 5.65 (s, 1H, benzyl-H), 3.70 (s, 12H, 4CH_3_), 2.89(q, 4H, *J* = 7.3 Hz, C*H*_2_CH_3_), 2.30(d, 4H, *J* = 14.7 Hz, CH_2_), 1.15(t, 6H, *J* = 7.3 Hz, CH_2_C*H*_3_), 1.10(s, 3H, CH_3_), 1.00(s, 3H, CH_3_); ^13^C NMR (100 MHz, CDCl_3_): *δ* = 161.6, 153.2, 145.5, 141.6, 129.1, 128.2, 127.8, 125.8, 88.5, 49.1, 41.9, 27.5, 11.5; Anal. for C_25_H_34_N_4_O_7_; calcd: C, 59.75; H, 6.82; N, 11.15; Found: C, 59.72; H, 6.80; N, 11.17; LC/MS (ESI): *m*/*z* = 502[M]^+^.

#### 5-((2-Hydroxy-4,4-dimethyl-6-oxocyclohex-1-en-1-yl)(4-(dimethylamino)phenyl)methyl)-1,3-dimethyl-2,6-dioxo-1,2,3,6-tetrahydropyrimidin-4-olate diethylaminium salt **4t**

**4t**; a beige material; m.p: 165 °C; (73 %, 550 mg, 1.1 mmol). IR (KBr, cm^−1^): 3055, 2950, 2865, 2500, 1669, 1580, 1510, 1427, 1373, 1255, 1214;^1^H NMR (400 MHz, CDCl_3_): *δ* 15.33 (s, 1H, OH), 7.02 (d, 2H, *J* = 8.0 Hz, Ph), 6.75 (d, 2H, *J* = 8.8 Hz, Ph), 5.69 (s, 1H, benzyl-H), 3.70 (s, 12H, 4CH_3_), 3.01 (s, 6H, N(CH_3_)_2_), 2.89(q, 4H, *J* = 7.3 Hz, C*H*_2_CH_3_), 2.31(d,4H, *J* = 14.7 Hz, CH_2_), 1.15(t, 6H, *J* = 7.3 Hz, CH_2_C*H*_3_), 1.12(s, 3H, CH_3_), 1.00(s, 3H, CH_3_); ^13^C NMR (100 MHz, CDCl_3_): *δ* = 161.6, 153.2, 145.5, 141.6, 129.1, 128.2, 127.8, 125.8, 88.5, 49.1, 41.9, 41.8, 27.5, 11.5; Anal. for C_27_H_39_N_4_O_5_; calcd: C, 64.91; H, 7.87; N, 11.21;Found: C, 64.90; H, 7.87; N, 11.23; LC/MS (ESI): *m*/*z* = 499.29[M]^+^.

#### 5-((2-Hydroxy-4,4-dimethyl-6-oxocyclohex-1-en-1-yl)(4-hydroxyphenyl)methyl)-1,3-dimethyl-2,6-dioxo-1,2,3,6-tetrahydropyrimidin-4-olate diethylaminium salt **4v**

**4v**; a white solid material; m.p: 162 °C; (91 %, 645 mg, 1.36 mmol). IR (KBr, cm^−1^): 23097, 2939, 2884, 2828, 2498, 1747, 1574, 1530, 1506, 1466, 1384, 1241;^1^H NMR (400 MHz, DMSO-*d*_6_): *δ* 14.52 (s, 1H, OH), 8.50 (brs, 1H, OH), 6.76(d, 2H, *J* = 8.0 Hz, Ph), 6.50(d, 2H, *J* = 8.0 Hz, Ph), 6.04(s, 1H, benzyl-H), 3.07 (s, 12H, 2CH_3_), 3.14(q, 4H, *J* = 7.3 Hz, C*H*_2_CH_3_), 2.92 (q, 4H, *J* = 13.9 Hz, CH_2_), 206 (s, 4H, CH_2_), 1.12(t, 6H, *J* = 7.3 Hz, CH_2_C*H*_3_), 0.98(s, 3H, CH_3_); ^13^C NMR (100 MHz, DMSO-*d*_6_): *δ* = 198.0, 188.5, 154.1, 136.6, 128.3, 115.3, 114.3, 90.1, 50.9, 45.5, 42.1, 31.6, 30.7, 29.7, 11.7; Anal. for C_25_H_35_N_3_O_6_; calcd: C, 63.41; H, 7.45; N, 8.87;Found: C, 63.40; H, 7.43; N, 8.85; LC/MS (ESI): *m*/*z* = 473 [M]^+^.

#### 4-((6-hydroxy-1,3-dimethyl-2,4-dioxo-1,2,3,4-tetrahydropyrimidin-5-yl)(2-hydroxy-4,4-dimethyl-6-oxocyclohex-1-en-1-yl)methyl)benzaldehyde diethylaminium salt **4x**

**4x**; as solid (1.26 g, 90 %). IR (cm^−1^): 3156, 2950, 2872, 1678, 1590, 1508, 1375, 1256, 1232, 1167; ^1^H-NMR (CDCl_3_, 400 MHz): 14.16 (s, 1H, OH), 9.80 (s, 1H, CHO), 8.01 (brs, 2H, NH), 6.98 (d, 2H, *J* = 7.3 Hz, Ph), 6.75 (d, 2H, *J* = 7.3 Hz, Ph), 5.61(s, 1H, benzyl-H), 3.73 (s, 6H, CH_3_), 2.92 (q, 4H, *J* = 7.3 Hz, C*H*_2_CH_3_), 2.31 (m, 4H, 2CH_2_), 1.26(t, 6H, *J* = 7.3 Hz, CH_2_C*H*_3_), 1.05(s, 3H, CH_3_), 1.00(s, 3H, CH_3_); ^13^C-NMR (100 MHz, CDCl_3_): *δ* = 193.0, 188.1, 165.0, 157.2, 127.8, 115.7,113.8, 91.6, 55.2, 48.8, 48.6, 42.4, 31.5, 29.4, 27.7, 11.7; Anal. for C_26_H_35_N_3_O_6_; Calcd: C, 64.31; H, 7.27; N, 8.65; Found:C, 64.30; H, 7.26; N, 8.63; LC/MS (ESI): *m/z* = 485.57 [M]^+^.

#### 5-((2,4-Dichlorophenyl)(2-hydroxy-4,4-dimethyl-6-oxocyclohex-1-en-1-yl)methyl)-1,3-dimethyl-2,6-dioxo-1,2,3,6-tetrahydropyrimidin-4-olate diethylaminium salt **4w**

**4w**; a beige solid material; m.p: 164 °C; (90 %, 710 mg, 1.35 mmol). IR (KBr, cm^−1^): 3059, 2995, 2867, 2114, 1741, 1658, 1591, 1463, 1429, 1370, 1341, 1256, 1201^1^H-NMR (400 MHz, CDCl_3_): *δ* 14.80 (s, 1H, OH), 7.29 (d, 1H, *J* = 8.0 Hz, Ph), 7.19 (s, 1H, Ph), 7.12(d, 2H, *J* = 8.0 Hz, Ph), 5.76 (s, 1H, benzyl-H), 3.28 (s, 12H, 4CH_3_), 3.07(q, 4H, *J* = 7.3 Hz, C*H*_2_CH_3_), 2.37 (s, 2H, CH_2_), 2.27 (d, 2H, *J* = 5.1 Hz, CH_2_), 1.34 (t, 6H, *J* = 7.3 Hz, CH_2_C*H*_3_), 1.04(s, 3H, CH_3_), 1.01 (s, 3H, CH_3_); ^13^C NMR (100 MHz, CDCl_3_): *δ* = 199.1, 165.4, 164.4, 152.5, 139.8, 133.6, 131.7, 131.2, 129.3, 126.4, 115.7, 89.8, 51.2, 45.7, 41.9, 32.4, 31.2, 28.3, 28.2, 11.3; Anal. for C_25_H_33_Cl_2_N_3_O_5_; calcd: C, 57.04; H, 6.32; Cl, 13.47; N, 7.98;Found: C, 57.09; H, 6.31; Cl, 13.44; N, 8.01; LC/MS (ESI): *m*/*z* = 526 [M]^+^.

#### 5-((2,6-Dichlorophenyl)(2-hydroxy-4,4-dimethyl-6-oxocyclohex-1-en-1-yl)methyl)-1,3-dimethyl-2,6-dioxo-1,2,3,6-tetrahydropyrimidin-4-olate diethylaminium salt **4y**

**4y** an oily material (89 %, 702 mg, 1.33 mmol). IR (KBr, cm^−1^): 3048, 2955, 2869, 2728, 2494, 1676, 1575, 1428, 1372, 1238, 1196;^1^H NMR (400 MHz, CDCl_3_): *δ* 14.80 (s, 1H, OH), 7.36 (d, 2H, *J* = 8.0 Hz, Ph), 7.29 (t, 1H, *J* = 8.0 Hz, Ph), 7.12(d, 2H, *J* = 8.0 Hz, Ph), 5.98 (s, 1H, benzyl-H), 3.26 (s, 12H, 4CH_3_), 2.92(q, 4H, *J* = 7.3 Hz, C*H*_2_CH_3_), 2.37 (s, 2H, CH_2_), 2.27 (d, 2H, *J* = 5.1 Hz, CH_2_), 1.24(t, 6H, *J* = 7.3 Hz, CH_2_C*H*_3_), 1.094(s, 3H, CH_3_), 1.04(s, 3H, CH_3_); ^13^C NMR (100 MHz, CDCl_3_): *δ* = 192.8, 188.9, 165.3, 164.3, 152.5, 149.7, 137.4, 131.5, 129.8, 126.5, 124.2, 115.5, 114.7, 89.9, 53.5, 41.4, 31.9, 28.7, 28.2, 11.4; Anal. for C_25_H_33_Cl_2_N_3_O_5_; calcd: C, 57.04; H, 6.32; Cl, 13.47; N, 7.98; Found: C, 57.08; H, 6.30; Cl, 13.45; N, 8.00; LC/MS (ESI): *m*/*z* = 526 [M]^+^.

#### 5-((2-Hydroxy-4,4-dimethyl-6-oxocyclohex-1-en-1-yl)(naphthalen-2-yl)methyl)-1,3-dimethyl-2,6-dioxo-1,2,3,6-tetrahydropyrimidin-4-olate diethylaminium salt **4z**

**4z**; a white solid material; m.p: 170 °C; (94 %, 715 mg, 1.41 mmol). IR (KBr, cm^−1^): 2994, 2948, 2866, 2506, 1742, 1651, 1603, 1570, 1526, 1473, 1431, 1362, 1245;^1^H NMR (400 MHz, CDCl_3_): *δ* 14.26 (s, 1H, OH), 7.46–7.22 (m, 7H, naphthyl), 6.20 (s, 1H, benzyl-H), 3.26 (s, 6H, 2CH_3_), 3.23 (s, 6H, 2CH_3_), 3.14(q, 4H, *J* = 7.3 Hz, C*H*_2_CH_3_), 2.41 (q, 4H, *J* = 5.1 Hz, CH_2_), 2.23 (s, 2H, CH_2_), 1.37(t, 6H, *J* = 7.3 Hz, CH_2_C*H*_3_), 1.07(s, 3H, CH_3_), 1.01(s, 3H, CH_3_); ^13^C NMR (100 MHz, CDCl_3_): *δ* = 199.0, 180.5, 165.3, 164.3, 152.5, 149.7, 136.8, 131.5, 129.9, 126.5, 124.2, 115.5, 114.7, 89.9, 50.9, 45.5, 41.7, 31.3, 30.7, 28.2, 11.1; Anal. for C_29_H_37_N_3_O_5_; calcd: C, 68.62; H, 7.35; N, 8.28; Found: C, 68.65; H, 7.34; N, 8.30; LC/MS (ESI): *m*/*z* = 507 [M]^+^.

#### 2-((2-Hydroxy-4,4-dimethyl-6-oxocyclohex-1-en-1-yl)(phenyl)methyl)-5,5-dimethyl-3-oxocyclohex-1-enolate diethylaminium salt **5a**

**5a**; as solid (1.26 g, 95 %). IR (cm^−1^): 2955 (s), 1586 (s), 1382 (s), 776 (s), 576 (s), 480 (s); ^1^H-NMR (CDCl_3_, 400 MHz) *δ* 13.91 (s, OH), 8.25 (bs, 1H. NH_2_), 7.01–7.21 (m, 5H. ArH), 5.74 (s, 1H, PhCH), 2.84 (q, *J* = 6.6 Hz, 4H, NHCH_2_CH_3_), 2.31 (s, 8H, CH_2_ + COCH_2_), 1.18 (t, *J* = 6.6 Hz, 6H, NHCH_2_CH_3_), 0.95–1.14 (m, 12H, CH_3_);^13^C-NMR (CDCl_3,_ 100 MHz): *δ* 199.1, 179.3, 142.4, 128.0, 126.8, 125.2, 115.5, 50.6, 45.9, 42.3, 34.2, 32.0, 11.4; Anal. Calcd.for C_27_H_37_NO_4_: C, 73.36; H, 8.98; N, 3.07; O, 14.57; Found: C, 73.43; H, 8.90; N, 3.17; O, 14.49; LC/MS (ESI): *m*/*z* = 441.29 [M]^+^.

#### 2-((4-Chlorophenyl)(2-hydroxy-4,4-dimethyl-6-oxocyclohex-1-en-1-yl)methyl)-5,5-dimethyl-3-oxocyclohex-1-enolate diethylaminium salt **5b**

**3c**; as solid (92 %, 1.31 g). IR (cm^−1^): 2956 (s), 1706 (s), 1573 (s), 1486 (s), 1382 (s), 1263 (s), 732 (s), 605 (s), 485 (s); ^1^H-NMR (CDCl_3_, 400 MHz) *δ* 13.59 (s, OH), 8.51 (bs, 2H. NH_2_), 6.89–7.21 (m, 4H. ArH), 5.70 (s, 1H, PhCH), 2.90 (q, *J* = 7.3 Hz, 4H, NHCH_2_CH_3_), 2.30 (s, 8H, CH_2_ + COCH_2_), 1.21 (t, *J* = 7.3 Hz, 6H, NH_2_CH_2_CH_3_), 0.91–1.16 (m, 12H, CH_3_); ^13^C-NMR (CDCl_3,_ 100 MHz): *δ*197.3, 188.6, 139.5, 130.8, 128.3, 128.1, 115.2, 49.7, 44.9, 42.2, 34.3, 33.1, 31.5, 11.3; Anal. Calcd. forC_27_H_38_ClNO_4_: C, 68.23; H, 8.19; N, 2.97; O, 13.34; Found: C, 68.12; H, 8.05; N, 2.90; O, 13.44; LC/MS (ESI): *m*/*z* = 475.25 [M]^+^.

#### 2-((2-Hydroxy-4,4-dimethyl-6-oxocyclohex-1-en-1-yl)(p-tolyl)methyl)-5,5-dimethyl-3-oxocyclohex-1-enolate diethylaminium salt **5c**

**5c**; as solid (93 %, 1.2 g). IR (cm^−1^): 2957 (s), 1571 (s), 1483 (s), 1383 (s), 1267 (s), 739 (s), 488 (s); ^1^H-NMR (CDCl_3_, 400 MHz) *δ* 13.73 (s, OH), 7.83 (bs, 2H. NH_2_), 6.91–7.05 (m, 4H. ArH), 5.73 (s, 1H, PhCH), 2.84 (q, *J* = 7.3 Hz, 4H, NHCH_2_CH_3_), 2.31 (s, 8H, CH_2_ + COCH_2_), 2.23 (s, 3H, PhCH_3_), 1.18 (t, *J* = 7.3 Hz, 6H, NH_2_CH_2_CH_3_), 0.94–1.16 (m, 12H, CH_3_), ^13^C-NMR (CDCl_3_, 100 MHz): *δ*195.8, 187.3, 144.4, 134.0, 128.6, 126.8, 115.6, 51.8, 46.1, 42.7, 34.9, 32.7, 31.4, 20.9,12.4; Anal. Calcd. forC_28_H_41_NO_4_: C, 73.79; H, 9.14; N, 3.09; O, 13.91; Found: C, 73.81; H, 9.07; N, 3.07; O, 14.05; LC/MS (ESI): *m*/*z* = 455.30 [M]^+^.

#### 2-((2-Hydroxy-4,4-dimethyl-6-oxocyclohex-1-en-1-yl)(m-tolyl)methyl)-5,5-dimethyl-3-oxocyclohex-1-enolate diethylaminium salt **5d**

**5d**; as solid (91 %, 1.24 g). IR (cm^−1^): 2952 (s), 1572 (s), 1483 (s), 1381 (s), 1227 (s), 1143 (s), 787 (s), 463 (s); ^1^H-NMR (CDCl_3_, 400 MHz) *δ* 13.78 (s, OH), 7.85 (bs, 2H. NH_2_), 6.88–7.03 (m, 4H. ArH), 5.71 (s, 1H, PhCH), 2.91 (q, *J* = 7.4 Hz, 4H, NHCH_2_CH_3_), 2.38 (s, 8H, CH_2_ + COCH_2_), 2.28 (s, 3H, PhCH_3_), 1.16 (t, *J* = 7.4 Hz, 6H, NH_2_CH_2_CH_3_), 0.91–1.12 (m, 12H, C**H**_3_); ^13^C-NMR (CDCl_3_, 100 MHz): *δ*195.9, 187.5, 144.7, 134.1, 128.4, 126.9, 115.8, 51.9, 46.3, 42.6, 34.8, 32.8, 31.2, 20.6, 12.3; Anal. Calcd.for C_28_H_41_NO_4_: C, 73.85; H, 9.09; N, 3.13; O, 13.79; Found: C, 73.81; H, 9.07; N, 3.07; O, 14.05; LC/MS (ESI): *m*/*z* = 455.30 [M]^+^.

#### 2-((2-Hydroxy-4,4-dimethyl-6-oxocyclohex-1-en-1-yl)(4-methoxyphenyl)methyl)-5,5-dimethyl-3-oxocyclohex-1-enolate diethylaminium salt **5e**

**5e**; as solid (89 %, 1.26 g). IR (cm^−1^): 3121 (s), 1668 (s), 1614 (s), 1578 (s), 1446 (s), 778 (s), 608 (s), 457 (s); ^1^H-NMR (CDCl_3_, 400 MHz) *δ* 14.67 (s, OH), 8.22 (bs, 2H. NH_2_), 6.97 (d, *J* = 7.4 Hz, 2H. ArH), 6.72 (d, *J* = 7.4 Hz, 2H, ArH), 5.72 (s, 1H, PhCH), 3.72 (s, 3H, OCH_3_), 2.85 (q, *J* = 7.4 Hz, 4H, NHCH_2_CH_3_), 2.30 (s, 8H, CH_2_ + COCH_2_), 1.20 (t, *J* = 7.4 Hz, 6H, NH_2_CH_2_CH_3_), 0.96–1.16 (m, 12H, CH_3_); ^13^C-NMR (CDCl_3_, 100 MHz): *δ* 194.1, 187.5, 157.6, 133.1, 127.8, 115.7, 113.4, 55.2, 50.7, 45.3, 42.5, 34.1, 31.5, 31.1,11.9; Anal. Calcd.for C_28_H_41_NO_5_: C, 71.19; H, 8.79; N, 3.05; O, 17.11; Found: C, 71.31; H, 8.76; N, 2.97; O, 16.96; LC/MS (ESI): *m*/*z* = 471.30 [M]^+^.

#### 2-((2-Hydroxy-4,4-dimethyl-6-oxocyclohex-1-en-1-yl)(4-nitrophenyl)methyl)-5,5-dimethyl-3-oxocyclohex-1-enolate diethylaminium salt **5f**

**5f**; as solid (90 %, 1.26 g). IR (cm^−1^): 2872 (s), 1582 (s), 1510 (s), 1466 (s), 1384 (s), 1339 (s), 757 (s), 487 (s); ^1^H-NMR (CDCl_3_, 400 MHz) *δ*15.12 (s, OH), 8.32(bs, 2H. NH_2_), 8.01 (m, *J* = 8.8 Hz, 2H.ArH), 7.21 (d, *J* = 8.8 Hz, 2H, ArH), 5.92 (s, 1H, PhCH), 2.94 (q, *J* = 7.3 Hz, 4H, NHCH_2_CH_3_), 2.29 (s, 8H, CH_2_ + COCH_2_), 1.21 (t, *J* = 7.3 Hz, 6H, NH_2_CH_2_CH_3_), 0.91–1.06 (m, 12H, CH_3_); ^13^C-NMR (CDCl_3_, 100 MHz): *δ* 194.9, 186.8, 151.9, 145.5, 127.7, 123.2, 114.8, 50.3, 42.5, 45.2, 34.1, 32.2, 31.6, 11.4; Anal. Calcd. forC_27_H_38_N_2_O_6_: C, 66.74; H, 7.98; N, 5.55; O, 19.91; Found: C, 66.64; H, 7.87; N, 5.76; O, 19.73; LC/MS (ESI): *m*/*z* = 468.27 [M]^+^.

#### 2-((2,6-Dichlorophenyl)(2-hydroxy-4,4-dimethyl-6-oxocyclohex-1-en-1-yl)methyl)-5,5-dimethyl-3-oxocyclohex-1-enolate diethylaminium salt **5g**

**5g**; as solid (91 %, 1.39 g). IR (cm^−1^): 2953 (s), 2869 (s), 1711 (s), 1575 (s), 1497 (s), 1367 (s), 1220 (s), 776 (s), 448 (s); ^1^H-NMR (CDCl_3_, 400 MHz) *δ* 14.78 (s, OH), 8.71 (bs, 2H. NH_2_), 7.24(s, *J* = 14.4 Hz, 1H, ArH), 7.16 (m, 1H, ArH), 6.95 (d, *J* = 14.4 Hz, 1H, ArH), 5.89 (s, 1H, PhCH), 2.90 (q, *J* = 7.4 Hz, 4H, NHCH_2_CH_3_), 2.19 (bs, 8H, CH_2_ + COCH_2_), 1.17 (t, *J* = 7.4 Hz, 6H, NH_2_CH_2_CH_3_), 0.88–1.03 (bs, 12H, CH_3_); ^13^C-NMR (DMSO-*d*_6_, 100 MHz): *δ* 198.3, 189.1, 139.1, 134.9, 128.2, 125.9, 114.2, 51.1, 47.6, 42.5, 34.3, 31.8, 30.3, 11.9; Anal. Calcd.for C_27_H_37_Cl_2_NO_4_: C, 63.46; H, 7.55; N, 2.43; O, 12.91; Found: C, 63.52; H, 7.31; N, 2.74; O, 12.54; LC/MS (ESI): *m*/*z* = 509.21 [M]^+^.

#### 2-((2-Hydroxy-4,4-dimethyl-6-oxocyclohex-1-en-1-yl)(3-nitrophenyl)methyl)-5,5-dimethyl-3-oxocyclohex-1-enolate diethylaminium salt **5h**

**5h**; as solid (90 %, 1.26 g). IR (cm^−1^): 2872 (s), 1582 (s), 1510 (s), 1466 (s), 1384 (s), 1339 (s), 757 (s), 487 (s); ^1^H-NMR (CDCl_3_, 400 MHz) *δ* 15.12 (s, OH), 8.32(bs, 2H. NH_2_), 8.01 (m, *J* = 8.8 Hz, 2H.ArH), 7.21 (d, *J* = 8.80 Hz, 2H, ArH), 5.92 (s, 1H, PhCH), 2.94 (q, *J* = 7.3 Hz, 4H, NHCH_2_CH_3_), 2.29 (s, 8H, CH_2_ + COCH_2_), 1.21 (t, *J* = 7.3 Hz, 6H, NH_2_CH_2_CH_3_), 0.91–1.06 (m, 12H, CH_3_); ^13^C-NMR (CDCl_3_, 100 MHz): *δ*194.9, 186.8, 151.9, 145.5, 127.7, 123.2, 114.8, 50.3, 45.2, 42.5, 34.1, 32.2, 31.6, 11.4; Anal. Calcd. forC_27_H_38_N_2_O_6_: C, 66.74; H, 7.98; N, 5.55; O, 19.91; Found: C, 66.64; H, 7.87; N, 5.76; O, 19.73; LC/MS (ESI): *m*/*z* = 468.27 [M]^+^.

#### 2-((2-Hydroxy-4,4-dimethyl-6-oxocyclohex-1-en-1-yl)(2-nitrophenyl)methyl)-5,5-dimethyl-3-oxocyclohex-1-enolate diethylaminium salt **5i**

**5i**; as solid (87 %, 1.22 g). IR (cm^−1^): 3096 (s), 2938 (s), 2869 (s), 1580 (s), 1539 (s), 1506 (s), 1384 (s), 1241 (s), 1033 (s), 778 (s), 604 (s), 524 (s); ^1^H-NMR (CDCl_3_, 400 MHz) *δ* 14.27 (s, OH), 8.74 (bs,2H. NH_2_), 7.10 (m, 4H, ArH), 6.22 (s, 1H, PhCH), 2.03 (q, *J* = 7.3 Hz, 4H, NHCH_2_CH_3_), 2.20 (bs, 8H, CH_2_ + COCH_2_), 1.29 (t, *J* = 7.3 Hz, 6H, NH_2_CH_2_CH_3_), 0.99 (bs, 12H, CH_3_);^13^C-NMR (CDCl_3_, 100 MHz): *δ* 198.9, 181.9, 149.7, 137.4, 131.3, 130.2, 125.9, 124.1, 114.5, 49.9, 44.8, 42.0, 33.6, 31.4, 29.4, 11.2; Anal. Calcd. forC_27_H_38_N_2_O_6_: C, 66.94; H, 7.87; N, 5.43; O, 19.96; Found: C, 66.64; H, 7.87; N, 5.76; O, 19.73; LC/MS (ESI): *m*/*z* = 468.27 [M]^+^.

#### 2-((4-Formylphenyl)(2-hydroxy-4,4-dimethyl-6-oxocyclohex-1-en-1-yl)methyl)-5,5-dimethyl-3-oxocyclohex-1-enolate diethylaminium salt **5j**

**5j**; as solid (75 %, 1.01 g). IR (cm^−1^): 3150 (s), 1586 (s), 1519 (s), 1469 (s), 1381 (s), 1339 (s), 779 (s), 495 (s); ^1^H-NMR (DMSO-*d*_6_, 400 MHz) *δ*16.45 (s, OH), 8.39 (bs, 2H. NH_2_), 6.78 (m, *J* = 8.04 Hz, 2H. ArH), 6.49 (d, *J* = 8.04 Hz, 2H, ArH), 6.08 (s, 1H, PhCH), 3.00 (s, 6H, N(CH_3_)_2_), 2.89 (q, *J* = 7.32 Hz, 4H, NHCH_2_CH_3_), 2.10 (s, 8H, CH_2_ + COCH_2_), 1.15 (t, *J* = 7.32 Hz, 6H, NH_2_CH_2_C**H**_3_), 0.88–1.01 (m, 12H, C**H**_3_); ^13^C-NMR (DMSO-*d*_6_,100 MHz): *δ*196.1, 183.6, 154.1, 136.1, 128.3, 115.3, 114.3,50.9,45.6, 42.0, 41.7, 34.2, 31.9, 29.8, 11.8; Anal. Calcd.for C_28_H_39_NO_5_: C, 71.61; H, 8.37; N, 2.98; Found: C, 71.61; H, 8.37; N, 2.98; LC/MS (ESI): *m*/*z* = 69.28 [M]^+^.

#### 2-((2-Hydroxy-4,4-dimethyl-6-oxocyclohex-1-en-1-yl)(4-hydroxyphenyl)methyl)-5,5-dimethyl-3-oxocyclohex-1-enolate diethylaminium salt **5k**

**5k**; as solid (88 %, 1.01 g). IR (cm^−1^): 3157 (s), 1584 (s), 1519 (s), 1469 (s), 1381 (s), 1339 (s), 779 (s), 495 (s); ^1^H-NMR (DMSO-*d*_6_, 400 MHz) *δ* 16.41 (s, OH), 8.32 (bs, 2H. NH_2_), 6.75 (m, *J* = 8.0 Hz, 2H. ArH), 6.45 (d, *J* = 8.0 Hz, 2H, ArH), 6.04 (s, 1H, PhCH), 2.88 (q, *J* = 7.3 Hz, 4H, NHCH_2_CH_3_), 2.50 (s, 1H, PhOH), 2.06 (s, 8H, CH_2_ + COCH_2_), 1.12 (t, *J* = 7.32 Hz, 6H, NH_2_CH_2_CH_3_), 0.85–0.97 (m, 12H, CH_3_); ^13^C-NMR (DMSO-*d*_6_, 100 MHz): *δ* 196.1, 183.6, 154.1, 136.1,128.3, 115.3, 114.3, 50.9, 45.6, 42.0, 34.2, 31.9, 29.8, 11.8; Anal. Calcd.for C_27_H_39_NO_5_: C, 70.74; H, 8.89; N, 3.13; O, 17.61; Found: C, 70.87; H, 8.59; N, 3.06; O, 17.48; LC/MS (ESI): *m*/*z* = 383.19 [M]^+^.

#### 4-((6-Hydroxy-1,3-dimethyl-2,4-dioxo-1,2,3,4-tetrahydropyrimidin-5-yl)(6-hydroxy-2,4-dioxo-1,2,3,4-tetrahydropyrimidin-5-yl)methyl)benzaldehyde diethylaminium salt **5l**

**5l**; as white solid (88 %, 1.20 g). IR (cm^−1^): 3455, 3305, 3000, 2910, 1677, 1582, 1510, 1466, 1384, 1339; ^1^H-NMR (CDCl_3_, 400 MHz) 17.30 (s, 1H, OH), 9.90 (s, 1H, CHO), 8.23 (brs, 2H, NH), 7.56 (d, 2H, *J* = 8.0 Hz, Ph), 7.11 (d, 2H, *J* = 8.0 Hz, Ph), 5.85(s, 1H, benzyl-H), 3.34 (s, 12H, 4CH_3_), 3.03 (q, 4H, *J* = 7.3 Hz, C*H*_2_CH_3_), 1.25 (t, 6H, *J* = 7.3 Hz, CH_2_C*H*_3_); ^13^C-NMR (100 MHz, CDCl_3_): *δ* = 192.1, 165.2, 164.1, 151.2, 150.0, 134.1, 129.5, 127.5, 91.6, 42.2, 35.1, 29.0, 28.7, 11.5; Anal. for C_22_H_27_N_5_O_7_; Calcd: C, 55.81; H, 5.75; N, 14.79; Found:C, 55.83; H, 5.76; N, 14.81; LC/MS (ESI): *m*/*z* = 473.48 [M]^+^.

#### 5-((4-Chlorophenyl)(2-hydroxy-4,4-dimethyl-6-oxocyclohex-1-en-1-yl)methyl)-2,6-dioxo-1,2,3,6-tetrahydropyrimidin-4-olate diethylaminium salt **5m**

**5m**; an oily product (90 %, 625 mg, 1.35 mmol). IR (KBr, cm^−1^): 3049, 2954, 2865, 2499, 1738, 1699, 1590, 1483, 1375, 1292, 1258, 1225, 1205;^1^H NMR (400 MHz, CDCl_3_): *δ* 13.32 (s, 1H, OH), 8.83 (brs, 2H, NH), 7.27(d, 2H, *J* = 8.0 Hz, Ph), 7.00(d, 2H, *J* = 8.0 Hz, Ph), 5.89 (s, 1H, benzyl-H), 2.88(q, 4H, *J* = 7.3 Hz, C*H*_2_CH_3_), 2.31 (d, 4H, *J* = 5.1 Hz, CH_2_), 1.19(t, 6H, *J* = 7.3 Hz, CH_2_C*H*_3_), 1.09(s, 3H, CH_3_), 1.03(s, 3H, CH_3_); ^13^C NMR (100 MHz, CDCl_3_): *δ* = 190.9, 141.0, 134.8, 131.0, 129.5, 128.3, 115.3, 91.1, 47.1, 42.7, 31.6, 31.5, 29.1, 28.2, 27.8, 11.3; Anal. for C_23_H_30_ClN_3_O_5_; calcd: C, 59.54; H, 6.52; Cl, 7.64; N, 9.06;Found: C, 59.57; H, 6.51; Cl, 7.60; N, 9.02; LC/MS (ESI): *m*/*z* = 463 [M]^+^.

#### 5-((2-Hydroxy-4,4-dimethyl-6-oxocyclohex-1-en-1-yl)(phenyl)methyl)-2,6-dioxo-1,2,3,6-tetrahydropyrimidin-4-olate diethylaminium salt **5n**

**5n**; a white solid material; m.p: 215 °C; (93 %, 598 mg, 1.39 mmol). IR (KBr, cm^−1^): 3027, 2948, 2867, 2156, 1683, 1593, 1451, 1374, 1291, 1257, 1141^1^H-NMR (400 MHz, CDCl_3_): *δ* 12.26 (s, 1H, OH), 9.31(brs, 2H, NH), 7.12(m, 5H, Ph), 5.52 (s, 1H, benzyl-H), 2.99(q, 4H, *J* = 7.3 Hz, C*H*_2_CH_3_), 2.45 (d, 4H, *J* = 5.1 Hz, CH_2_), 1.24(t, 6H, *J* = 7.3 Hz, CH_2_C*H*_3_), 1.09(s, 3H, CH_3_), 1.03(s, 3H, CH_3_); ^13^C NMR (100 MHz, CDCl_3_): *δ* = 198.5, 180.8, 152.5, 142.5, 128.0, 126.7, 125.1, 116.3, 90.9, 51.4, 45.9, 42.2, 33.0, 28.4, 27.6, 11.3; Anal. for C_23_H_31_N_3_O_5_; calcd: C, 64.32; H, 7.27; N, 9.78;Found: C, 64.29; H, 7.29; N, 9.80; LC/MS (ESI): *m*/*z* = 429[M]^+^.

#### 5-((4-Bromophenyl)(2-hydroxy-4,4-dimethyl-6-oxocyclohex-1-en-1-yl)methyl)-2,6-dioxo-1,2,3,6-tetrahydropyrimidin-4-olate diethylaminium salt **5o**

**5o**; a white solid material; m.p: 208 °C; (89 %, 678 mg, 1.33 mmol); IR (KBr, cm^−1^): 3093, 2939, 2885, 2829, 2551, 1746, 1686, 1576, 1506, 1466, 1416, 1268, 1241; ^1^H NMR (400 MHz, CDCl_3_): *δ* 13.31 (s, 1H, OH), 8.67 (brs, 2H, NH), 7.05(m, 4H, Ph), 5.79 (s, 1H, benzyl-H), 2.79(q, 4H, *J* = 7.3 Hz, C*H*_2_CH_3_), 2.35 (d, 4H, *J* = 5.1 Hz, CH_2_), 1.21(t, 6H, *J* = 7.3 Hz, CH_2_C*H*_3_), 1.11(s, 3H, CH_3_), 1.03(s, 3H, CH_3_); ^13^C NMR (100 MHz, CDCl_3_): *δ* = 198.5, 180.1, 152.8, 140.5, 131.4, 130.7, 128.7, 128.6, 118.5, 115.6, 91.0, 50.9, 42.8, 31.6, 31.5, 29.2, 28.3, 27.8, 11.3; Anal. for C_23_H_30_BrN_3_O_5_; calcd: C, 54.34; H, 5.95; Br, 15.72; N, 8.27;Found: C, 54.35; H, 5.96; Br, 15.69; N, 8.30; LC/MS (ESI): *m*/*z* = 508 [M]^+^.

#### 5-((2-Hydroxy-4,4-dimethyl-6-oxocyclohex-1-en-1-yl)(p-tolyl)methyl)-2,6-dioxo-1,2,3,6-tetrahydropyrimidin-4-olate diethylaminium salt **5p**

**5p**; a white solid material; m.p: 213 °C; (91 %, 604 mg, 1.36 mmol). IR (KBr, cm^−1^): 3150, 2955, 2867, 1690, 1592, 1508, 1375, 1256, 1232, 1167;^1^H NMR (400 MHz, CDCl_3_): *δ* 13.31 (s, 1H, OH), 8.83 (brs, 2H, NH), 7.27(d, 2H, *J* = 8.0 Hz, Ph), 7.00(d, 2H, *J* = 8.0 Hz, Ph), 5.88 (s, 1H, benzyl-H), 2.83(q, 4H, *J* = 7.3 Hz, C*H*_2_CH_3_), 2.31 (d, 4H, *J* = 5.1 Hz, CH_2_), 2.23 (s, 3H, CH_3_), 1.19(t, 6H, *J* = 7.3 Hz, CH_2_C*H*_3_), 1.04(s, 3H, CH_3_), 1.02(s, 3H, CH_3_); ^13^C NMR (100 MHz, CDCl_3_): *δ* = 196.5, 180.1, 152.8, 140.5, 131.4, 130.7, 128.7, 128.6, 118.5, 115.6, 91.0, 50.9, 42.8, 31.6, 31.5, 29.2, 28.3, 27.8, 20.9, 11.3; Anal. for C_24_H_33_N_3_O_5_; calcd: C, 64.99; H, 7.50; N, 9.47;Found: C, 64.95; H, 7.49; N, 9.50; LC/MS (ESI): *m*/*z* = 443 [M]^+^.

#### 2-((4-Formylphenyl)(6-hydroxy-2,4-dioxo-1,2,3,4-tetrahydropyrimidin-5-yl)methyl)-5,5-dimethyl-3-oxocyclohex-1-enolate diethylaminium salt **5q**

**5q**; a white solid material; m.p: 205 °C; (87 %, 594 mg, 1.3 mmol). IR (KBr, cm^−1^): 3145, 2950, 2870, 1677, 1550, 1510, 1375, 1256, 1232, 1167; ^1^H NMR (400 MHz, CDCl_3_): *δ* 13.35 (s, 1H, OH), 9.92 (s, 1H, CHO), 8.80 (brs, 2H, NH), 7.30(d, 2H, *J* = 8.0 Hz, Ph), 7.05(d, 2H, *J* = 8.0 Hz, Ph), 5.85 (s, 1H, benzyl-H), 2.89(q, 4H, *J* = 7.3 Hz, C*H*_2_CH_3_), 2.30 (d, 4H, *J* = 5.1 Hz, CH_2_), 2.26(s, 3H, CH_3_), 1.22(t, 6H, *J* = 7.3 Hz, CH_2_C*H*_3_), 1.08(s, 3H, CH_3_), 1.05(s, 3H, CH_3_); ^13^C NMR (100 MHz, CDCl_3_): *δ* = 198, 181.3, 152.8, 140.5, 131.4, 130.7, 128.7, 128.6, 118.5, 115.6, 91.0, 50.9, 42.8, 31.6, 31.5, 29.2, 28.3, 27.8, 20.9, 11.3; Anal. for C_24_H_31_N_3_O_6_; calcd: C, 63.00; H, 6.83; N, 9.18;Found: C, 63.01; H, 6.84; N, 9.18; LC/MS (ESI): *m*/*z* = 457 [M]^+^.

#### 5-((2-Hydroxy-4,4-dimethyl-6-oxocyclohex-1-en-1-yl)(naphthalen-2-yl)methyl)-2,6-dioxo-1,2,3,6-tetrahydropyrimidin-4-olate diethylaminium salt **5r**

**5r**; an oily product (90 %, 646 mg, 1.35 mmol). IR (KBr, cm^−1^): 3049, 2948, 2863, 2725, 1685, 1594, 1508, 1371, 1252, 1216; ^1^H NMR (400 MHz, CDCl_3_): *δ* 14.25 (s, 1H, OH), 7.46-7.22(m, 7H, naphthyl), 6.21 (s, 1H, benzyl-H), 3.27 (s, 6H, 2CH_3_), 3.25 (s, 6H, 2CH_3_), 3.14(q, 4H, *J* = 7.3 Hz, C*H*_2_CH_3_), 2.41 (q, 4H, *J* = 5.1 Hz, CH_2_), 2.23 (s, 2H, CH_2_), 1.37(t, 6H, *J* = 7.3 Hz, CH_2_C*H*_3_), 1.07(s, 3H, CH_3_), 1.01(s, 3H, CH_3_); ^13^C NMR (100 MHz, CDCl_3_): *δ* = 199.1, 180.5, 165.5, 164.2, 152.5, 149.7, 136.8, 131.5, 129.9, 126.5, 124.2, 115.5, 114.7, 89.9, 50.9, 45.5, 41.7, 31.3, 30.7, 28.2, 11.3; Anal. for C_27_H_33_N_3_O_5_; calcd: C, 67.62; H, 6.94; N, 8.76; Found: C, 67.65; H, 6.96; N, 8.80; LC/MS (ESI): *m*/*z* = 479 [M]^+^.

#### 2-((2-Hydroxy-4,4-dimethyl-6-oxocyclohex-1-en-1-yl)(naphthalen-2-yl)methyl)-5,5-dimethyl-3-oxocyclohex-1-enolate diethylaminium salt **5s**

**5s**; as solid (93 %, 1.33 g). IR (cm^−1^): 3053 (s), 2943 (s), 2866 (s), 1688 (s), 1566 (s), 1511 (s), 1383 (s), 1241 (s), 1035 (s), 774 (s), 482 (s), 554 (s); ^1^H-NMR (CDCl_3_, 400 MHz) *δ* 1.01 (bs, 12H, C**H**_3_), 1.19 (t, *J* = 7.3 Hz, 6H, NH_2_CH_2_CH_3_), 2.29 (bs, 8H, CH_2_ + COCH_2_), 2.88 (q, *J* = 7.3 Hz, 4H, NHCH_2_CH_3_), 6.32 (s, 1H, PhCH), 7.55–7.64 (m, 2H, ArH), 7.69 (t, *J* = 7.4 Hz, 1H, ArH), 7.91 (d, *J* = 8.8 Hz, 1H, ArH), 7.99 (d, *J* = 6.6 Hz, 1H, ArH), 8.10 (d, *J* = 8.1 Hz, 1H, ArH), 9.25 (d, *J* = 8.0 Hz, 1H, ArH), 1039 (s,2H. NH_2_), 14.25 (s, OH); ^13^C-NMR (CDCl_3_, 100 MHz): *δ* 193.6, 182.8, 136.8, 135.4, 133.8, 131.5, 124.7, 116.8, 50.5, 130.6, 128.6, 129.1, 127.0, 45.3, 42.2, 33.9, 31.4, 29.8, 11.7; Anal. Calcd.for C_30_H_39_NO_4_: C, 75.83; H, 8.05; N, 3.03; O, 13.29; Found: C, 75.71; H, 8.23; N, 2.91; O, 13.40; LC/MS (ESI): *m*/*z* = 477.29 [M]^+^.

#### Procedure for In vitro Urease Inhibiton Assay

Reaction mixture comprising of 25μL of enzyme (jack bean urease) (1 unit/well) solution and 55 μL of phosphate buffers (4 mM) containing 100 mM urea were incubated with 5 μL of test compounds dissolved in methanol (0.5 mM concentration) at 30 °C for 15 min in 96-well plates. Urease activity was determined by measuring ammonia production using the indophenol method as described by Weather burn [30]. Briefly, 45 μl each phenol reagent (1 % w/v phenol and 0.005 % w/v sodium nitroprussside) and 70 μL of alkali reagent(0.5 % w/v NaOH and 0.1 % active chloride NaOCl) were added to each well. The increasing absorbance at 630 nm was measured afther 50 min, using a microplate reader (Molecular Device, USA). All reactions were performed in triplicate in a final volume of 200 μL. The results (change in absorbance per min) were processed by using softMax Pro software (molecular Device, USA). The entire assays were performed at pH 6.8. Percentage inhibitions were calculated from the formula 100 − (OD_testwell_/OD_control_) × 100. Thiourea was used as the standard inhibitor of urease [31, 32].

### Materials and methods for MD simulation and molecular docking studies

#### Receptor and ligand preparation

The crystal structure of helicobacter pylori (HP) urease in complex with acetohydroxamic acid, (PDB entry code 1E9Y) was retrieved from the protein data bank [33]. All the water molecules were removed from the PDB crystal structure and hydrogen atoms were added. This structure was followed by energy minimization with amber99 force field (http://www.chempcomp.com) in the molecular operating environment (MOE) Software packages [34]. The three dimensional structure of the compounds were constructed via Builder module implemented in MOE. Subsequently all the compounds structures were minimized by using MMFF94 force field [35] in MOE preceding to molecular docking studies.

#### Protocol selection

Initially docking was performed for both the isomers i.e. keto and enol form. For docking purpose, default docking parameters of MOE is used such as Triangle Matcher Algorithm with two different rescoring functions. London dG and GBVI/WSA dG were used to generate 30 poses of each ligand and were saved in MOE database. Finally, docking results were analyzed by visualizing several interactions of compounds within binding pocket of proteins.

#### Molecular dynamic simulation

The keto and enol complexes were energy-minimized to eliminate possible steric strain up to 0.1 gradients by using AMBER99 force field. The relaxed complexes were then subjected to MD simulations using MOE 2013.0801 software. Each complex was gradually simulated at 300 K for 100 ps, in order to simulate the physiological conditions, system is allowed to maintain at physiological temperature of 300 K. The temperature is attained gradually, to avoid protein destruction, over a period of 100 ps. Initially, protein is heated from 0 to 50 K, followed by its ramping to 100, 200 and finally 300 K and then equilibrated at 300 K for even distribution of water molecules keeping protein molecule constrained. After equilibration step MD simulation was performed for 5 ns by using the Nose-Poincare-Anderson (NPA) method [36]. To make ensemble trajectories NVT ensemble was used.

The trajectory output files were saved after every 1 ps for future analysis. Equilibration was monitored by convergence in terms of the temperature, energy, density and the RMSD (root-mean-squared deviations) of the backbone atoms as compared to the crystal structure of both complexes.

## Results and discussion

### Chemistry

**Scheme 1:**
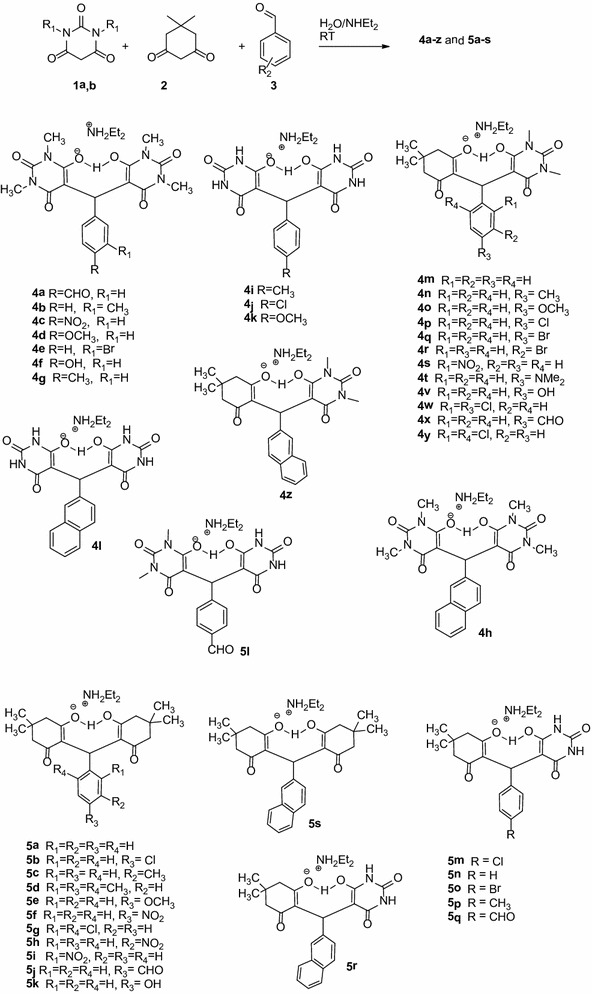
Synthesis of compounds **4a**–**z** and **5a**–**s**

In our continued interest [30, 37–47] in the development of highly expedient methods for the synthesis of diverse heterocyclic compounds of biological importance via one-pot multi-component reactions (MCRs) and avoiding organic solvents during the reactions in organic synthesis leads to efficient, environmentally benign reagents, clean, and economical technology (Green Chemistry Concepts). In the present investigation, reaction of equimolar amounts of barbituric acid **1a,b** dimedone **2** with aldehyde **3** in presence of aqueous diethylamine medium at RT afforded zwitterionic adducts **4a**–**z** and **5a**–**s** in quantitative yields by simple filtration (Scheme 1).

### Biological activity

Thirty-two new derivatives of barbituric acid as zwitterionic adducts of diethyl ammonium salts having *bis*(6-hydroxy-1,3-dimethyl-2,4-dioxo-1,2,3,4-tetrahydropyrimidin-5-yl) (**4a**–**h**), *bis*-(6-hydroxypyrimidine-2,4(1*H*,3*H*)-dione) (**4i**–**4l**), (2-hydroxy-4,4-dimethyl-6-oxocyclohex-1-en-1-yl)-1,3-dimethyl-2,6-dioxo-1,2,3,6-tetrahydropyrimidin-4-olate (**4m**–**4z**), 4-((6-Hydroxy-1,3-dimethyl-2,4-dioxo-1,2,3,4-tetrahydropyrimidin-5-yl)(6-hydroxy-2,4-dioxo-1,2,3,4-tetrahydropyrimidin-5-yl)methyl) benzaldehyde (**5l**), (2-hydroxy-4,4-dimethyl-6-oxocyclohex-1-en-1-yl)methyl-2,6-dioxo-1,2,3,6-tetrahydropyrimidin-4-olate (**5m**–**5r**) and twelve derivatives of dimedone as zwitterionic adducts of diethyl ammonium salts having *bis*-(2-hydroxy-4,4-dimethyl-6-oxocyclohex-1-en **(5a**–**k** and **5s)** as basic nucleus were screened in vitro for their ureas enzyme inhibition potential against thiourea (IC_50_ = 21.2 ± 1.3 µM), as an standard tested compounds (Table 1).

**Table 1:** In vitro urease inhibiton activity of compounds **4a**–**z** and **5a**–**s**

Compound	Urease inhibition IC_50_ ± SEM [µM]	Compound	Urease inhibition IC_50_ ± SEM [µM]
**4a**	39.3 ± 0.36	**4x**	38.5 ± 0.28
**4b**	34.4 ± 1.57	**4y**	83.4 ± 1.00
**4c**	54.2 ± 0.47	**4z**	39.8 ± 1.38
**4d**	31.6 ± 0.79	**5a**	74.5 ± 0.88
**4e**	27.5 ± 0.12	**5b**	29.7 ± 0.67
**4f**	54.2 ± 0.83	**5c**	61.4 ± 1.12
**4g**	28.5 ± 0.41	**5d**	51.3 ± 0.45
**4h**	40.3 ± 0.32	**5e**	39.8 ± 0.75
**4i**	17.6 ± 0.23	**5f**	106.4 ± 1.49
**4j**	22.3 ± 0.73	**5g**	170.7 ± 1.55
**4k**	25.8 ± 0.23	**5h**	49.0 ± 0.55
**4l**	22.7 ± 0.20	**5i**	210.1 ± 0.29
**4m**	39.3 ± 0.79	**5j**	72.6 ± 0.59
**4n**	41.2 ± 0.58	**5k**	43.8 ± 0.33
**4o**	83.0 ± 0.66	**5l**	17.2 ± 0.44
**4p**	39.7 ± 0.70	**5m**	65.9 ± 0.61
**4q**	24.6 ± 0.42	**5n**	23.7 ± 0.57
**4r**	27.5 ± 0.19	**5o**	34.6 ± 0.79
**4s**	109.7 ± 1.10	**5p**	27.4 ± 0.54
**4t**	142.1 ± 0.64	**5q**	41.6 ± 0.41
**4v**	52.2 ± 1.26	**5r**	82.8 ± 0.72
**4w**	59.4 ± 0.98	**5s**	123.2 ± 0.37
STD. Thiourea	21.2 ± 1.3		

Among barbituric acid zwitterionic adducts (**4a**–**h**) having *bis*(6-Hydroxy-1,3-dimethyl-2,4-dioxo-1,2,3,4-tetrahydropyrimidin-5-yl) ring as basic nucleus, all ccompounds **4a**, **4b**, **4d**, **4e**, **4g** and **4f** showed IC_50_ values 39.3 ± 0.36, 34.4 ± 1.57, 31.6 ± 0.79, 27.5 ± 0.12, 28.5 ± 0.41, and 40.3 ± 0.32 µM respectively, and were found to be the potent urease inhibitors except compounds **4c** (IC_50_ = 54.2 ± 0.47 µM) and **4f** (IC_50_ = 54.2 ± 0.83 *µ*M), while compared with the standard compound thiourea (IC_50_ = 21.2 ± 1.3 µM).

Among the barbituric acid derived derivatives (**4i**–**4l**), having *bis*(6-hydroxypyrimidine-2,4(1H,3H)-dione) as backbone, all tested compounds *i.e.***4i** (IC_50_ = 17.6 ± 0.23 µM), **4j** (IC_50_ = 22.3 ± 0.73 µM), **4** **k** (IC_50_ = 25.8 ± 0.23 µM) and **4** **l** (IC_50_ = 22.7 ± 0.20 µM) were found to be potent inhibitors of urease enzyme. Methyl substituted phenyl ring containing compound **4i** (IC_50_ = 17.6 ± 0.23 µM) was the most active candidate of the series.

Third series of the derivatives of barbituric acids having (2-Hydroxy-4,4-dimethyl-6-oxocyclohex-1-en-1-yl)-1,3-dimethyl-2,6-dioxo-1,2,3,6-tetrahydropyrimidin-4-olate ring as basic nucleus (**4m**–**4z**) were also evaluated for their urease enzyme inhibition. Compounds **4m** (IC_50_ = 39.3 ± 0.79 µM), **4n** (IC_50_ = 41.2 ± 0.58 µM), **4p** (IC_50_ = 39.7 ± 0.70 µM), **4q** (IC_50_ = 24.6 ± 0.42 µM), **4r** (IC_50_ = 27.5 ± 0.19 µM), **4x** (IC_50_ = 38.5 ± 0.28 µM), and **4z** (IC_50_ = 39.8 ± 1.38 µM) was found to be potent urease inhibitors against the standard thiourea.

Among fourth series of the derivatives of barbituric acid having (2-hydroxy-4,4-dimethyl-6-oxocyclohex-1-en-1-yl)methyl-2,6-dioxo-1,2,3,6-tetrahydropyrimidin-4-olate) ring as basic nucleus (**5m**–**5r**), compound **5n** (IC_50_ = 23.7 ± 0.57 µM), **5o** (IC_50_ = 34.6 ± 0.79 µM), **5p** (IC_50_ = 27.4 ± 0.54 µM), and **5q** (IC_50_ = 41.6 ± 0.41 µM), showed poetnt urease inhibiton. All other compounds found to be weak urease inhibitors.

Similarly dimedone derivatives, *bis*-(*2*-*hydroxy*-*4,4*-*dimethyl*-*6*-*oxocyclohex*-*1*-*en*) ring conatining compounds **5a**–**s** were also evaluated for their in vitro urease enzyme inhibition potential. Compounds **5b** (IC_50_ = 29.7 ± 0.67 µM), **5e** (IC_50_ = 39.8 ± 0.75 µM), and **5k** (IC_50_ = 43.8 ± 0.33 µM), showed good enzyme inhibtion. All other compounds found to be significant to weak urease inhibitors (IC_50_ = 49.0 ± 0.55–210.1 ± 0.29 µM).

On the basis of the evaluated urease inhibition abilities of the above five different series of barbituric acid and dimedone derivatives as zwitter ion adduct compounds **4i** (IC_50_ = 17.6 ± 0.23 µM) and **5** **l** (IC_50_ = 17.2 ± 0.44 µM) found to be the most active compounds and showed more urease inhibiton poetntial than the standard compound thiourea.

### Molecular modeling and docking studies

In order to obtain deep insight into the binding mechanism of barbituric acid derivatives within the active site of urease enzyme and to obtain further validations of experimental results, MD simulation studies were performed.

**Fig. 1:**
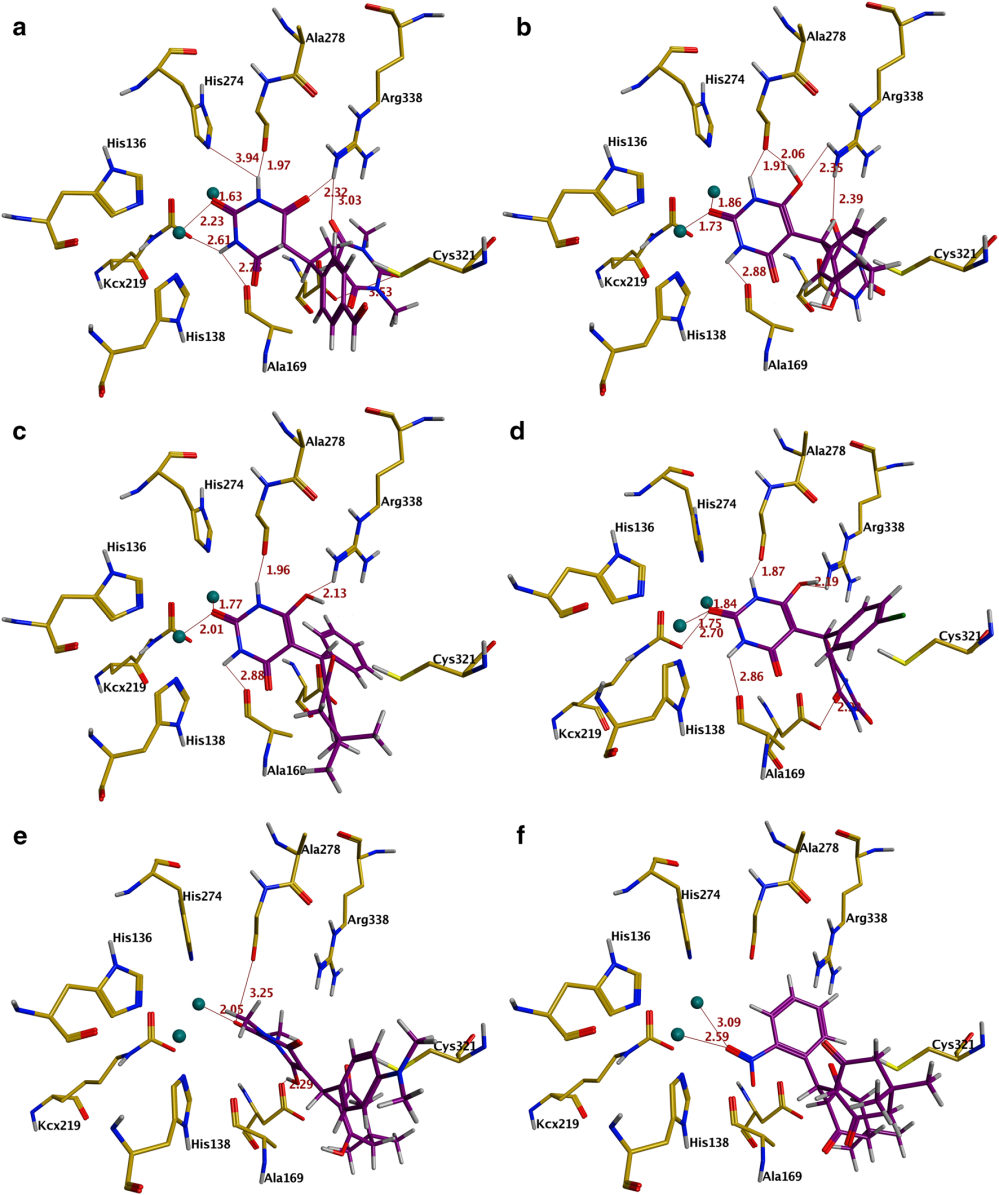
The docked poses of urease inhibitors: most active **5l** (**a**), **4i** (**b**), active **5n** (**c**), **4j** (**d**) and least active **4t** (**e**), **5g** (**f**). The interacting residues are presented in *yellow stick* while the ligands are shown in *purple sticks*

Forty-four barbituric acid derivatives (1–44) were docked into the binding pocket of urease. All the compounds were observed to accept analogous conformations with similar binding mode around the binding site of urease and these compounds were found to interact with nickel metal ions and the hotspot binding pocket residues (His137, His138, Ala169, KCX219, Asp362, Ala366 etc.) Visual inspection for predicted binding conformations of most potent compounds **5l** and **4i** (IC50 = 17.2 ± 0.44 M and IC_50_ = 17.6 ± 0.23 M) revealed that both compounds can adopt conformation for a better fit into the binding groove of urease. Further analysis of the top ranked poses of these compounds revealed that these compounds involved in multiple hydrogen bonding interactions with His138, Ala169, KCX219, Gly279, Asp362 and Arg338 residues. Compound **5i** was found to be the least potent among the active ligands with IC_50_value of 210.1 ± 0.29 *μ*M. Additionally; compounds **4a**–**4h**, **4j**–**4r**, and **4v**–**5e**, **5m**–**5q**, **5h** and **5k** showed a good urease inhibitory activity. In case of most active compound **5l** the amine moiety adjacent to the carbonyl group formed hydrogen bond with the side chain oxygen of modified lysine KCX 219 at a distance of 2.61 Å. Another hydrophillic interaction found between carbonyl moiety of aldehyde group at para position of benzene ring and NH of His323 at a distance of 2.82 Å. The compound is further stabilized by the numerous hydrophilic interactions provided by catalytic residues Ala169 (2.75 Å), Gly279 (1.97 Å) and Asp362 (3.14 Å) NH moiety with carbonyl oxygen of compound **5l**. Another important residue Arg338 formed two hydrogen bonds with carbonyl oxygen of pyrimidine ring at a distance of 2.32 and 3.03 Å, respectively. The best docked conformation of compound **5l** predicted by MOE showed that the compound is deeply inserted within the urease binding site which is further stabilize by multiple hydrophilic and hydrophobic interactions, contributing to the higher activity of this compound. The binding mode of **5l** is represented in Fig. 1a.

The binding mechanism of compound **4i** reveals that multiple hydrogen bonding interactions found between ligand and hotspot residues. The carbonyl of pyrimidine moiety engaged in hydrogen bonding interaction with the NH of KCX219 and Arg338 at a distance of 2.71 and 2.35 Å, respectively. The NH of pyrimidine ring form hydrogen bond with the oxygen of Ala169, Gly279 and Asp362 at a distance of 2.88, 1.91 and 3.38 Å, respectively. Moreover these interactions are further stabilized by polar interactions with the His138. Due to the absence of aldehyde group at *meta* position of compound **4i**, it is unable to make hydrophillic interaction with His323. The binding pattern of **4i** in the ligand binding site of urease is shown in Fig. 1b.

Compounds **4j** and **5n** adopted a similar binding mechanism to **5l** with some minor changes. The carbonyl oxygen of these compounds are hydrogen bonded to NH of Arg338 (2.19 and 2.13 Å) and KCX219 (2.7 and 2.91 Å), while NH of pyrimidine moiety of these compounds are engaged in hydrogen bond interactions with Ala169 (2.86 and 2.88 Å), Gly279 (1.87 and 1.96 Å) and Asp 362 (3.01 and 3.33 Å) as observed in **5l** (Fig. 1). The non substituted benzene ring of the molecule is exposed to the surface and not found to be involved in such interactions. The docked orientation of **4j** and **5n** is shown in Fig. 1c, d.

To explain the inactivity of compounds **4s**–**4t**, **5f**–**5g**, **5i** and **5s** all the inactive compounds were also docked in the urease binding cavity by using MOE. By docking pose analysis, it is evident that all the inactive compounds were poorly occupied in the binding site. The carbonyl oxygen of **4t**, **5g** and **5i** is replaced by an alkyl group as a result, hydrogen bonding interactions with catalytic residues are lost. The docked poses are shown in Fig. 1e, f. Similarly, the compounds **4s**, **5i** containing nitro group at R1 position instead of electron donating group may be one of the reasons for the inactivity of these compounds. These compounds interact with nickel ions but interactions with the catalytic residues are not as effective as observed in case of active compounds and shown in Fig. 1e, f). Consequently, absence of hydrogen bond interactions of these inactive compounds with crucial residues Ala169, Arg338 and Asp362 might be the reason for the inactivity of these compounds in the in vitro assay.

### Molecular dynamic simulations

**Fig. 2:**
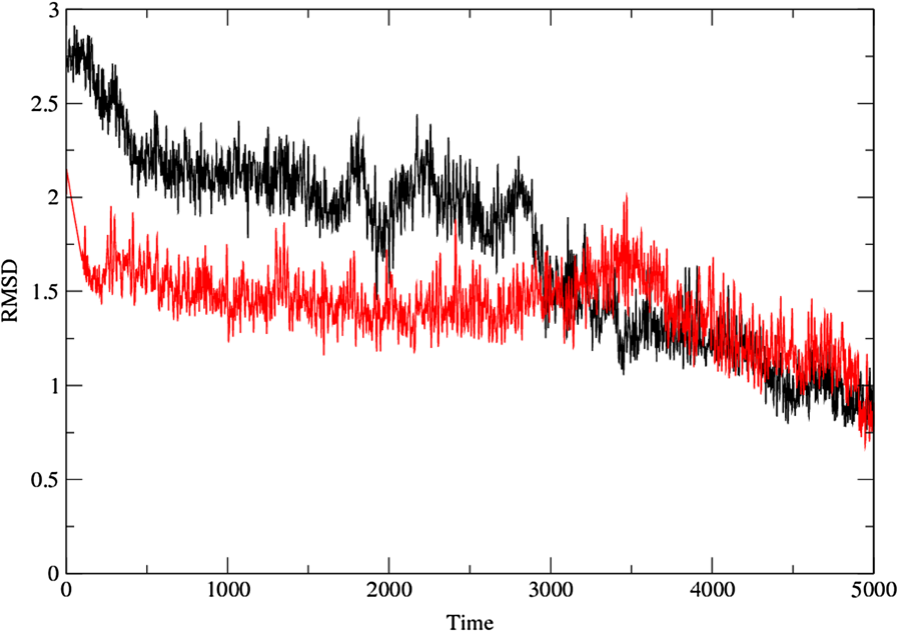
The RMSD plot of barbituric acid derivatives enol and keto complexes. *Red color* represents the enol form and *black color* represents the keto form RMSD

In order to understand the binding mechanism of barbituric acid derivatives molecular dynamic (MD) simulation was performed. The enol form of barbituric acid derivatives is found to be more stable during MD simulation as compared to its keto form. The keto form established only a weak interaction with nickel as compared to the enol. Interaction with nickel ion is crucial for inhibitory mechanism of urease inhibitors. The two forms disagree after 500 ps simulation as the distance between Ni and keto form increases gradually. To obtain further interaction pattern for two different form of barbituric acid derivatives, docking was also performed with both the possible forms. In the docking experiment, similar interactions with catalytic residues were observed except interaction with nickel metal. The obtained conformation explained the three dimensional structure of protein, which can be changed without fluctuating covalent bonds. The RMSD plot of protein conformation verses time for both the complexes is given in Fig. 2, which support the stability of enol form as nickel complex.

## Conclusion

This study conclude that a simple one step chemistry can generate extra-ordinary array bioactive compounds. During this study, we synthesized barbituric acid derivatives by simple filtration and evaluated for their urease inhibitory activity. Compounds (**4a**–**4z** and **5a**–**s**) were evaluated for their urease inhibition potential in vitro against the standard compound thiourea (IC_50_ = 128.8 ± 2.1 µM). Compounds **4i** (IC_50_ = 17.6 ± 0.23 µM) and **5l** (IC_50_ = 17.2 ± 0.44 µM) were found to be the most active members of the series with several fold more urease inhibition activity than the standard compound thiourea. The promising result of the current study indicates that barbituric acid derivatives can be investigated for the treatment of urease associated complications, such as peptic ulcer.

## Additional file

10.1186/s13065-015-0140-1 Supplementary information containing the spectra of the synthesized compounds.
