# Supplementary material for: Synthesis and dynamics studies of barbituric acid derivatives as urease inhibitors
Source: Chem Cent J. 2015 Nov 17;9:63. doi: 10.1186/s13065-015-0140-1 (PMC4648982; doi:10.1186/s13065-015-0140-1)

**Synthesis and Dynamics Studies of Barbituric Acid Derivatives as Urease Inhibitors**

**Assem Barakat^1,2^*^†^, Abdullah Mohammed Al-Majid^1†^, Gehad Lotfy^3†^, Fiza Arshad^4†^, Sammer Yousuf^4†^, M. Iqbal Choudhary^1,4,5†^ Sajda Ashraf^5†^ and** [**Zaheer Ul-Haq**](https://www.researchgate.net/researcher/15380316_Zaheer_Ul-Haq)**^5†^**

^1^Department of Chemistry, College of Science, King Saud University, P.O. Box 2455,
Riyadh 11451, Saudi Arabia.

^2^Department of Chemistry, Faculty of Science, Alexandria University, P.O. Box 426- Ibrahimia, Alexandria 21321, Egypt.

^3^Organic Chemistry Department, Faculty of Pharmacy, Suez Canal University, Ismailia, Egypt.

^4^H.E.J. Research Institute of Chemistry, International Center for Chemical and Biological Sciences, University of Karachi, Karachi-75270, Pakistan.

^5^Dr. Panjwani Center for Molecular Medicine and Drug Research, International Center for Chemical and Biological Sciences, University of Karachi, Karachi-75270, Pakistan.

*^†^Equal contributors.*

******* *Author to whom correspondence should be addressed; E-Mail:* [*ambarakat@ksu.edu.sa*](mailto:ambarakat@ksu.edu.sa)*; Tel.: +966-11467-5884; Fax: +966-11467-5992.*

AMA: amajid@ksu.edu.sa

GA: [lotfygehad@yahoo.com](mailto:lotfygehad@yahoo.com)

FA: Arshad@hotmail.com

SY: dr.sammer.yousuf@gmail.com

MIC: iqbalhej@yahoo.com

SA: Ashrafsajda@hotmail.com

ZU-H: zaheer_qasmi@hotmail.com

**Table of contents**

The ORTEP generated plot of the X-ray and H&C-NMRs of some selected compounds

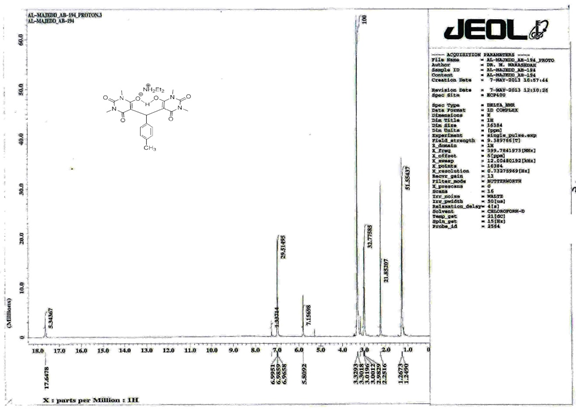


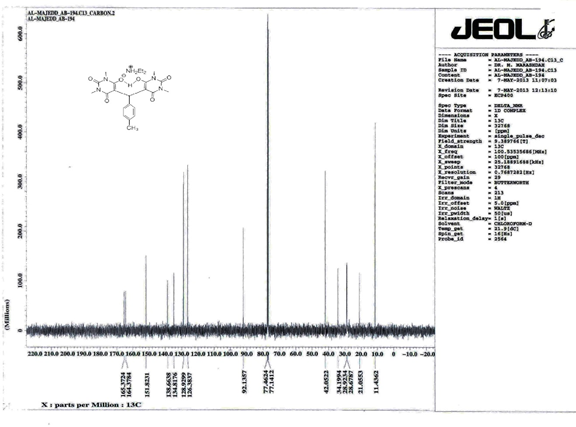


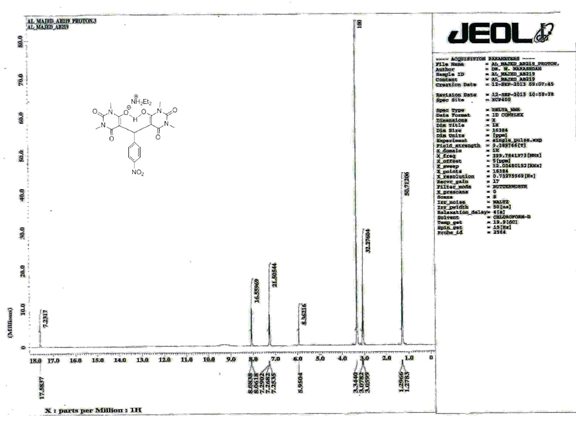


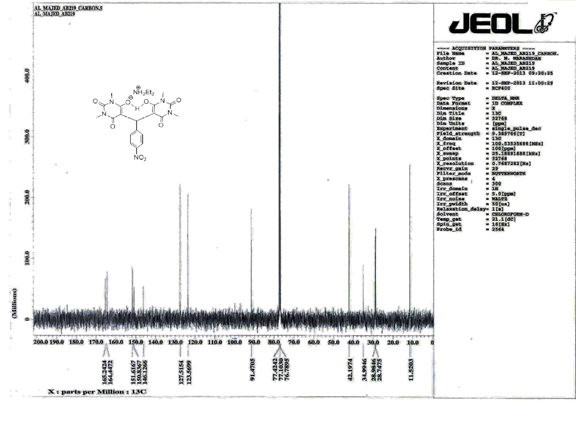


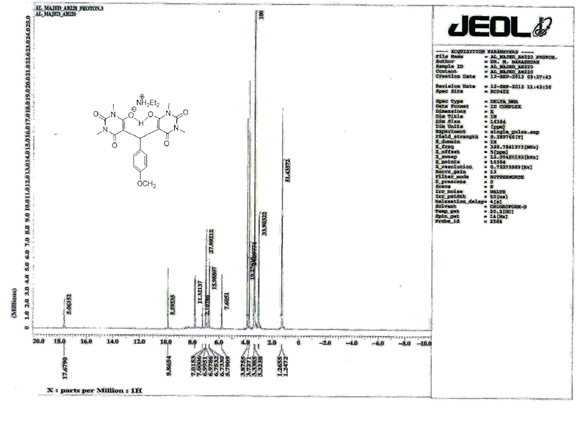


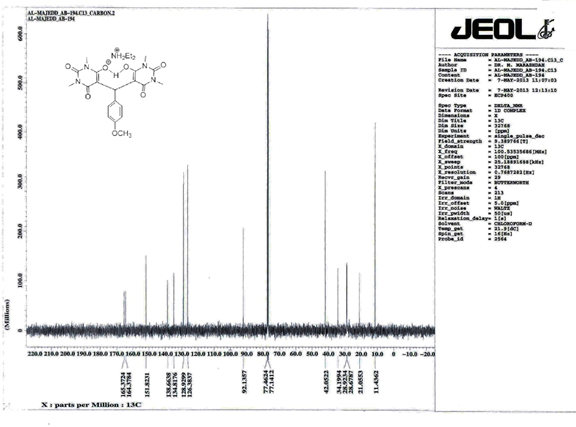


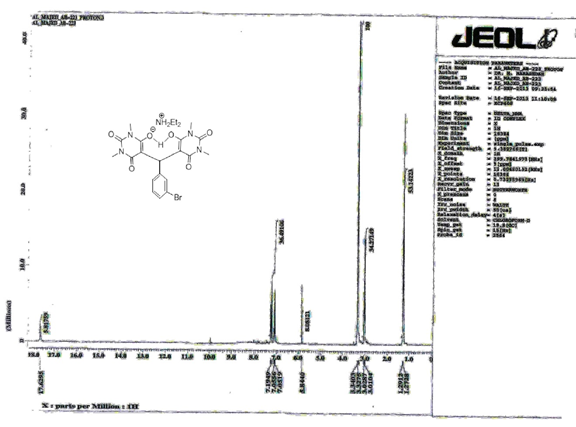


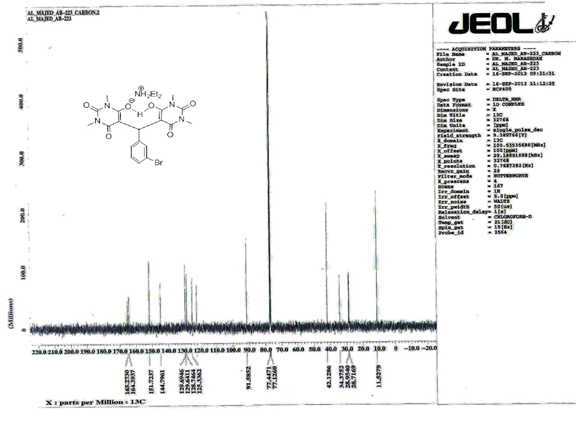


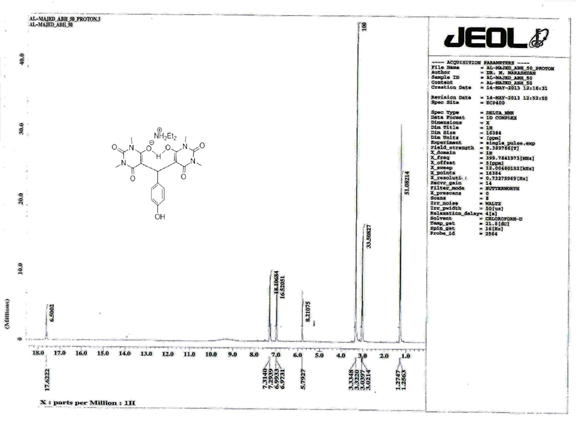


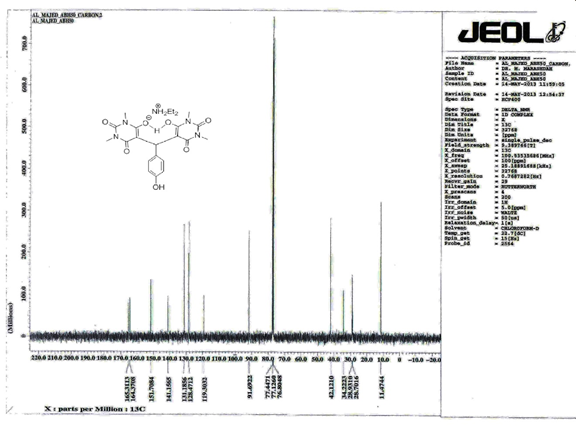


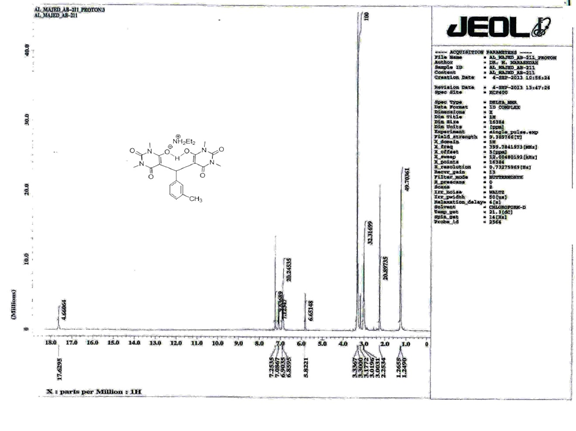


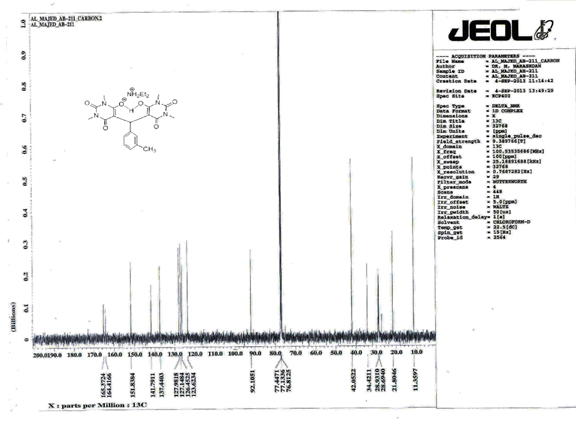


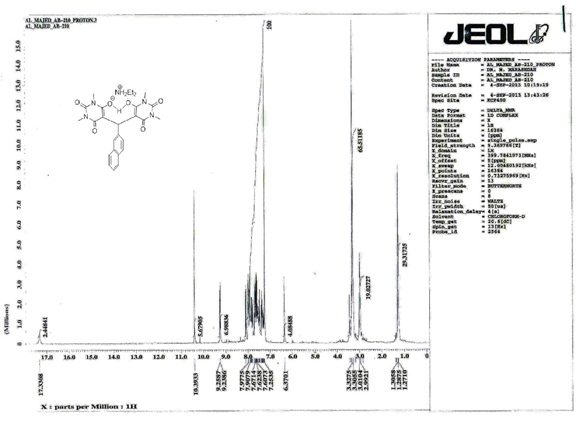


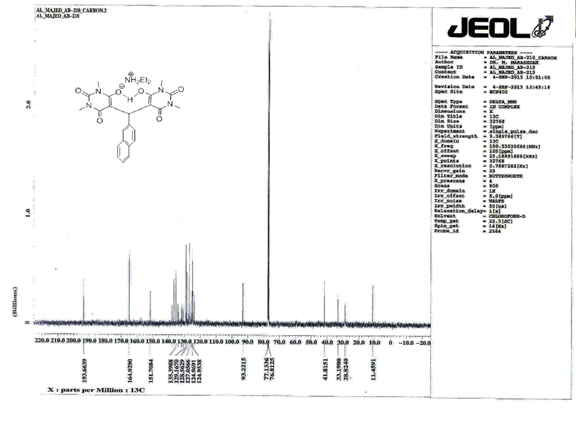


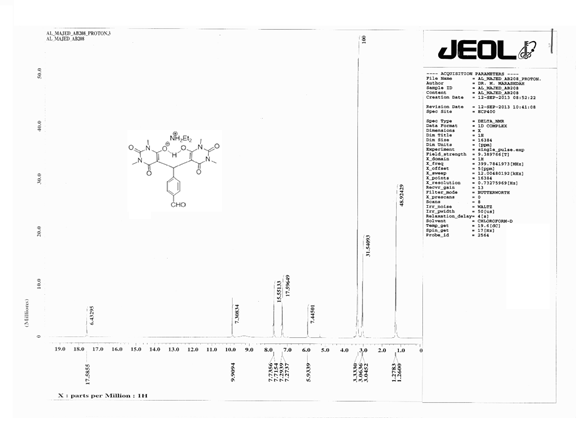


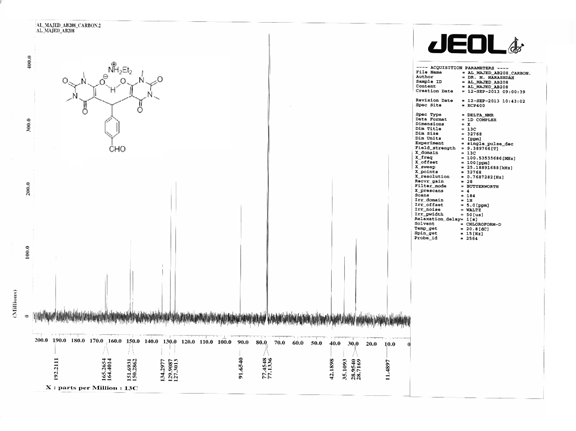


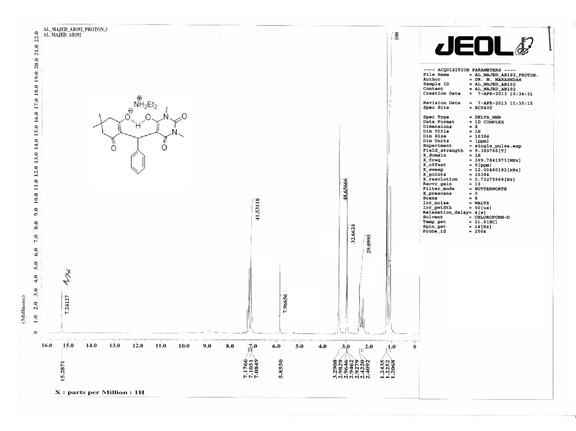


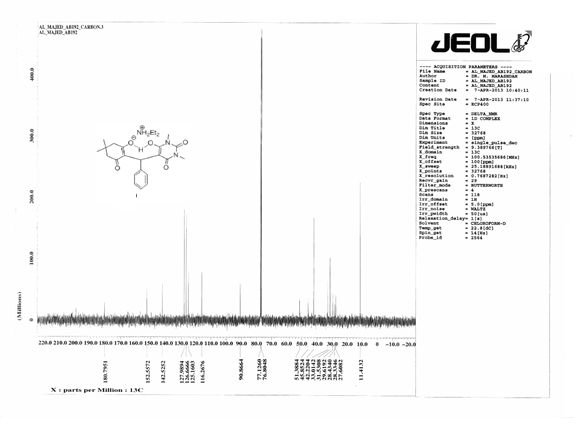


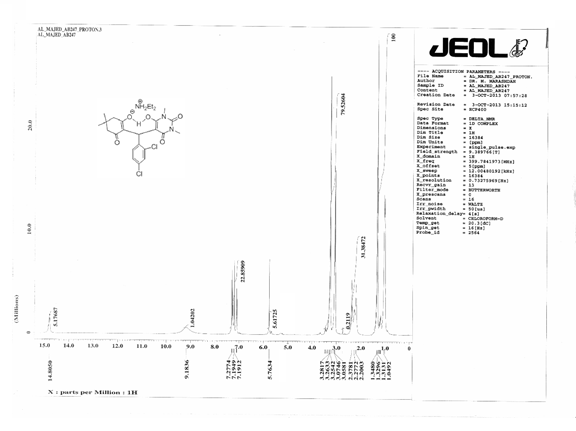


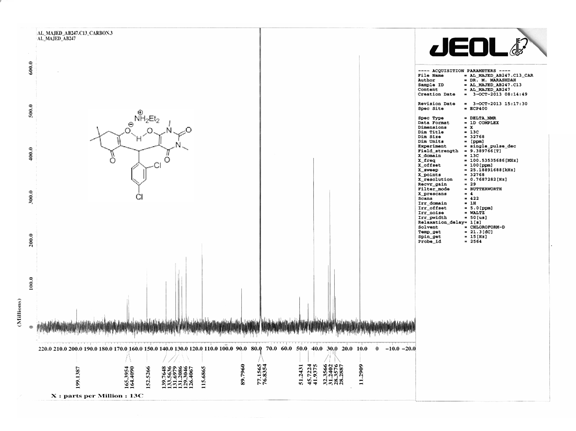


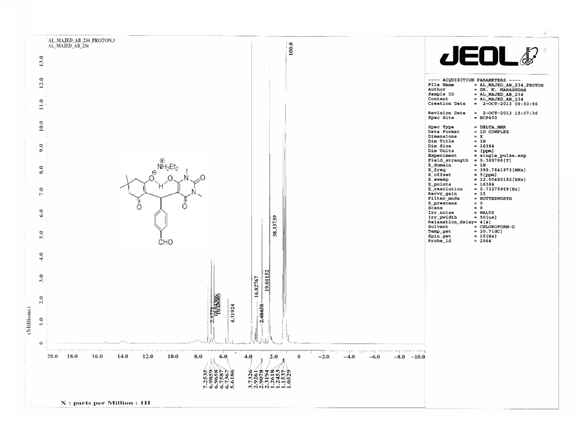


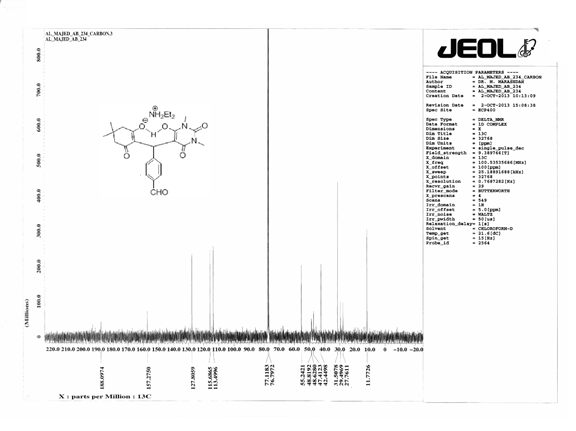


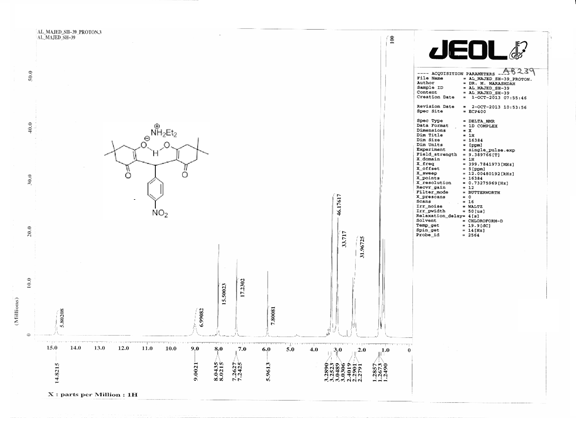


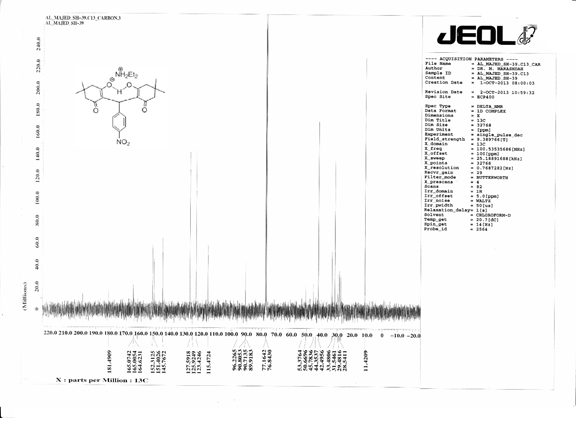

Supplement: Supplementary file 1 — 10.1186/s13065-015-0140-1 Supplementary information containing the spectra of the synthesized compounds. [file 13065_2015_140_MOESM1_ESM.docx]
